# Large-scale transcriptome sequencing in broiler chickens to identify candidate genes for breast muscle weight and intramuscular fat content

**DOI:** 10.1186/s12711-021-00656-9

**Published:** 2021-08-16

**Authors:** Huimin Kang, Di Zhao, Hai Xiang, Jing Li, Guiping Zhao, Hua Li

**Affiliations:** 1 Guangdong Provincial Key Laboratory of Animal Molecular Design and Precise Breeding; Key Laboratory of Animal Molecular Design and Precise Breeding of Guangdong Higher Education Institutes; School of Life Science and Engineering, Foshan University, #33 Guang-yun-lu, Shishan, Nanhai, Foshan, 528231 Guangdong People’s Republic of China; 2 Institute of Animal Sciences, Chinese Academy of Agricultural Sciences, No. 2 Yuanmingyuan West Road, Beijing, 100193 People’s Republic of China; 3 Guangdong Tinoo’s Foods Group Co., Ltd, Jiangkou, Feilaixia, Qingcheng, Qingyuan, 511827 Guangdong People’s Republic of China

## Abstract

**Background:**

In broiler production, breast muscle weight and intramuscular fat (IMF) content are important economic traits. Understanding the genetic mechanisms that underlie these traits is essential to implement effective genetic improvement programs. To date, genome-wide association studies (GWAS) and gene expression analyses have been performed to identify candidate genes for these traits. However, GWAS mainly detect associations at the DNA level, while differential expression analyses usually have low power because they are typically based on small sample sizes. To detect candidate genes for breast muscle weight and IMF contents (intramuscular fat percentage and relative content of triglycerides, cholesterol, and phospholipids), we performed association analyses based on breast muscle transcriptomic data on approximately 400 Tiannong partridge chickens at slaughter age.

**Results:**

First, by performing an extensive simulation study, we evaluated the statistical properties of association analyses of gene expression levels and traits based on the linear mixed model (LMM) and three regularized linear regression models, i.e., least absolute shrinkage and selection operator (LASSO), ridge regression (RR), and elastic net (EN). The results show that LMM, LASSO and EN with tuning parameters that are determined based on the one standard error rule exhibited the lowest type I error rates. Using results from all three models, we detected 43 candidate genes with expression levels that were associated with breast muscle weight. In addition, candidate genes were detected for intramuscular fat percentage (1), triglyceride content (2), cholesterol content (1), and phospholipid content (1). Many of the identified genes have been demonstrated to play roles in the development and metabolism of skeletal muscle or adipocyte. Moreover, weighted gene co-expression network analyses revealed that many candidate genes were harbored by gene co-expression modules, which were also significantly correlated with the traits of interest. The results of Gene Ontology and Kyoto Encyclopedia of Genes and Genomes enrichment analyses indicated that these modules are involved in muscle development and contraction, and in lipid metabolism.

**Conclusions:**

Our study provides valuable insight into the transcriptomic bases of breast muscle weight and IMF contents in Chinese indigenous yellow broilers. Our findings could be useful for the genetic improvement of these traits in broiler chickens.

**Supplementary Information:**

The online version contains supplementary material available at 10.1186/s12711-021-00656-9.

## Background

Chicken and pork are the dominant meats consumed around the world. Global meat consumption is expected to increase as populations continue to grow, as is per person consumption of meat. White broiler chickens are the main type of chicken raised for consumption and their selection is mainly aimed on fast growth for breast muscle weight [1], which has improved by nearly 50% since the beginning of the twenty-first century [2]. Chinese indigenous yellow broilers are increasingly favored by consumers because of their good meat quality. In spite of their slower growth rate, they account for 38% of the chicken meat production in China [3]. Further enhancements to their breeding programs could further improve their breast muscle weight. In addition to weight, intramuscular fat (IMF) content in meat has also received much more attention in recent years. It is a major determinant of meat quality [4] that contributes to flavor, tenderness and juiciness [5, 6]. IMF quantifies the amount of lipids, i.e. triglycerides (TG), cholesterol (CHO), and phospholipids (PL) [7], that are deposited in the muscle, both between and within muscle fibers. Chicken meat with a high IMF content has gained popularity with consumers.

Given the importance of breast muscle weight and IMF content, it is essential to understand their genetic bases to implement effective genetic improvement programs. With the development of high-density single nucleotide polymorphism (SNP) chips, genome-wide association studies (GWAS) have been performed to detect associations of SNPs with breast muscle weight [8–11] and with IMF percentage [8, 12, 13]. Based on the Animal Quantitative Trait Loci (QTL) database (accessed Feb 2021) [14], approximately 150, 25, 15, and 19 QTL have been identified in chickens for breast muscle weight, IMF percentage, TG level, and CHO level, respectively. Although these studies have advanced our understanding of the genetic bases of these traits, the mechanisms by which many of the identified QTL and SNPs act remain largely unknown. Gene expression analyses can provide a snapshot of actively expressed genes and transcripts under various conditions. Differentially expressed genes (DEG) have been uncovered for breast muscle weight and lipid contents using differential expression analysis in chickens [7, 15–18]. While significantly associated SNPs are, in many cases, located in intergenic regions, the biological functions of DEG for the analyzed traits can be explored more directly. However, differential expression analyses usually involve a small number of individuals, which explains their low detection power and poor reproducibility [19]. Furthermore, most traits of interest in animal breeding are quantitative traits. Differential expression analyses reveal genes that are differentially expressed in individuals that belong to different groups, e.g., a fast-growing group and a slow-growing group. In this case, quantitative traits are analyzed as categorical traits and their phenotypic information is not fully exploited in the analysis. However, similar to GWAS for quantitative traits that are performed with SNPs, association analyses based on gene expression levels and phenotypic records can be carried out to detect candidate genes for traits of interest. In addition to association studies, weighted gene co-expression network analysis (WGCNA) is widely used in gene expression analyses to explore the correlation patterns among expression levels of genes and to associate gene co-expression network modules to traits [20], including breast muscle IMF [21].

In the current study, we collected breast muscle samples from approximately 400 Tiannong partridge chickens at slaughter age for RNA-seq. The Tiannong partridge chicken is a commercially used three-way cross chicken produced using three pure lines of Qingyuan partridge chicken, which is an important Chinese indigenous slow-growing yellow broiler and is well-known for its superior meat quality. We also measured their breast muscle weights and IMF contents (IMF percentage and relative content of TG, CHO and PL). Association analyses were performed between gene expression levels and breast muscle-related traits to identify candidate genes for these traits. The optimal strategy to use the RNA-seq data in the association analysis was identified using a simulation study. Gene co-expression network modules for candidate genes were investigated using WGCNA. To the best of our knowledge, this is the first large-scale transcriptome-wide investigation of breast muscle weight and IMF contents in chickens. The results not only facilitate the study of molecular genetic mechanisms underlying these traits but may also lay the foundation for their genetic improvement.

## Methods

### Animals and sampling

In total, 399 female Tiannong partridge chickens were obtained from Guangdong Tinoo’s Foods Group Co., Ltd. All birds were raised in one of the company’s farms from 1 to 125 days of age, following the commercial feeding standard. The chickens were randomly sampled from those raised in the farm and slaughtered on day 126 using a mechanized slaughter line with moderate scalding water (61 °C). Then, the left and right breast muscles of each bird were separated from the bones and weighed and the combined weight was used as the phenotype for breast muscle weight. Phenotypic values on 381 birds were accurately recorded and used in subsequent analyses. Pectoral muscle samples were dissected from the same area of each chicken, snap-frozen using dry ice, and stored at − 80 °C for subsequent RNA isolation and measurement of biochemical indices.

### Measurement of biochemical indices

IMF content of each pectoral muscle sample was determined as crude fat using Soxhlet extraction (PN-ISO 1444: 2000) with fat solvents (Soxtherm SOX 406, Gerhardt) [22]. The TG and CHO contents in pectoral muscle samples were measured using TG and CHO assay kits (Nanjing Jiancheng Bioengineering Institute, Nanjing, China). A sample of pectoral muscle (about 2 g) from each chicken was homogenized with absolute ethanol at room temperature and centrifuged (1000×*g*, 20 min). After centrifugation, the supernatant was used for TG and CHO measurement. A 2.5-μL aliquot of the supernatant and 250 μL of working reagent were co-incubated at 37 °C for 10 min. The absorbance of each sample was measured using a Tecan Infinite 200 microplate reader at 510 nm according to the manufacturer’s instructions. The PL content of each sample was evaluated using a Chicken PL ELISA Kit (Shanghai Enzyme Union Biotechnology Co., Ltd, Shanghai, China) by measuring the absorbance of each sample using a Tecan Infinite 200 microplate reader at 490 nm. Finally, PL level was calculated based on a typical standard curve.

### RNA extraction and sequencing

Total RNA of a sample of pectoral muscle from each chicken was isolated using TRIzol reagent (Invitrogen, Carlsbad, CA, USA) and its quality was confirmed as described in [23]. RNA purity was assessed using the kaiaoK5500^®^ Spectrophotometer (Kaiao, Beijing, China) and RNA integrity and concentration were assessed using the RNA Nano 6000 Assay Kit with the Bioanalyzer 2100 system (Agilent Technologies, CA, USA). RNA samples with an A260/A280 ratio between 1.8 and 2.0 and an RNA integrity number higher than 7.5 were used for RNA sequencing.

The cDNA library was constructed according to the procedure described by Chen et al. [24]. mRNA samples were enriched by binding the mRNA poly-A tail to magnetic beads with Oligo (dT) and fragmented into small pieces. Using mRNA as a template, single- and double-stranded cDNA were synthesized. The double-stranded cDNA was purified using the QIAQuick PCR purification kit (QIAGEN, Valencia, CA, USA). After purification, end repair, and ligation to sequencing adapters, we used agarose gel electrophoresis for fragment size selection. Finally, PCR enrichment was performed to obtain the final cDNA library. RNA-sequencing was performed on an Illumina NovaSeq 6000 (Illumina, San Diego, CA, USA) and 150 bp paired-end reads were generated.

### Phenotypic analysis

Means and standard deviations for breast muscle weight and IMF contents were calculated using the R functions mean and sd, respectively. Pearson correlations between traits were tested using the R function cor.test, which also estimated the 95% confidence intervals for correlations. A hierarchical clustering dendrogram for breast muscle-related traits was generated using the R functions dist and hclust, with default values.

### Data analysis of RNA sequencing

The software program FastQC [25] was used to assess the quality of raw sequence data. The sequence adapters were trimmed using the BBMap software [26] and then reads were filtered with the fastp program [27]. Sequenced reads were aligned to the chicken reference genome [GRCg6a (GCA_000002315.5)] using the HISAT2 program [28]. We used the program featureCounts [29] to count the number of reads that mapped to each gene and the input files were prepared using the samtools software [30]. Read counts were normalized using the DESeq2 software [31].

We performed WGCNA using the standard method [20]. For each module, we calculated eigengene values for gene expression and subsequently tested if there was a significant correlation (*p* < 0.05) between the eigengene expression value and the traits analyzed. Gene ontology (GO) enrichment analysis and Kyoto Encyclopedia of Genes and Genomes (KEGG) pathway enrichment analysis were performed for the significantly correlated modules using clusterProfiler [32], with the *p*-value adjusted by the Bonferroni correction method, and *p*- and *q*-value cutoffs of 0.05. For each co-expression network of interest, hub genes were identified by the maximal clique centrality (MCC) algorithm, which was reported to be the most effective method of finding hub nodes [33]. The MCC of each gene was calculated by CytoHubba, a plugin in Cytoscape [33]. Genes with the top 10% MCC values were considered as hub genes.

### Statistical models for association analyses

To select the most appropriate strategy for detecting associations between gene expression levels and traits, the performance of four commonly used linear regression models was evaluated using simulated data, i.e., linear mixed model (LMM), least absolute shrinkage and selection operator (LASSO), ridge regression (RR), and elastic net (EN). Then, candidate genes for traits related to breast muscle were detected using LMM, LASSO and EN with tuning parameters determined based on the one standard error rule (based on the results of the simulation study). All data, including phenotypic records and gene expression levels, were scaled to a mean of zero and one unit of variance prior to association analyses for both the simulation and the empirical studies.

First, we evaluated the single locus LMM, in which the association between gene expression and the trait under study is tested one gene at a time, using the following model for the vector of phenotypes for the trait:

1 $$\mathbf{y}=\mu \mathbf{1}+{b}_{i}{\mathbf{X}}_{i}+\mathbf{a}+\mathbf{e},$$

where $\mu$ is the population mean, **1** is a vector of 1s, the independent variable ${\mathbf{X}}_{i}$ is a vector of expression values of the $i$th gene, and ${b}_{i}$ is the effect of the $i$th gene on the trait under study, $\mathbf{a}$ is a vector of the random polygenic effects, assumed distributed $N\left(\mathbf{0},\mathbf{K}{\sigma }^{2}\right)$, where ${\sigma }^{2}$ is the variance of this random effect, and $\mathbf{K}$ is the covariance structure inferred from all transcriptome data as follows:

2 $$\mathbf{K}=\frac{1}{d}\sum_{i=1}^{m}{\mathbf{X}}_{i}{\mathbf{X}}_{i}^{\mathrm{^{\prime}}},$$

where $m$ is the number of genes and $d$ is the average value of the diagonal of the matrix $\sum_{i=1}^{m}{\mathbf{X}}_{i}{\mathbf{X}}_{i}^{^{\prime}}$. The $\mathbf{K}$ matrix is analogous to the kinship matrix that is used in GWAS to capture genetic relationships among individuals. The residual error is assumed to be normally distributed as $N\left(\mathbf{0},\mathbf{I}{\sigma }_{e}^{2}\right)$.

The other three models investigated for association analyses were LASSO [34], RR [35], and EN [36]. These are multi-locus models, in which the expression levels of all genes are analyzed jointly. LASSO, RR, and EN are classified as regularized linear regression models, for which the basic linear regression model is:

3 $$\mathbf{y}=\mu \mathbf{1}+\sum_{i=1}^{m}{\mathbf{X}}_{i}{b}_{i}+\mathbf{e}.$$

The LASSO estimator obtains a sparse solution using ${l}_{1}$ penalized least squares:

4 $$\widehat{\mathbf{b}}\left(LASSO\right)=\mathrm{arg}\underset{\mathbf{b}}{\mathrm{min}}\left\{{\left(\mathbf{y}-\mu \mathbf{1}-\mathbf{X}\mathbf{b}\right)}^{\mathrm{^{\prime}}}\left(\mathbf{y}-\mu \mathbf{1}-\mathbf{X}\mathbf{b}\right)+\lambda \sum_{i=1}^{m}\left|{b}_{i}\right|\right\},$$

where $\lambda$ is the tuning parameter obtained via cross-validation.

The RR estimator solves this regression problem using ${l}_{2}$ penalized least squares:

5 $$\widehat{\mathbf{b}}\left(RR\right)=\mathrm{arg}\underset{\mathbf{b}}{\mathrm{min}}\left\{{\left(\mathbf{y}-\mu \mathbf{1}-\mathbf{X}\mathbf{b}\right)}^{\mathrm{^{\prime}}}\left(\mathbf{y}-\mu \mathbf{1}-\mathbf{X}\mathbf{b}\right)+\lambda \sum_{i=1}^{m}{b}_{i}^{2}\right\}.$$

The EN model uses a mixture of ${l}_{1}$ and ${l}_{2}$ penalties and can be formulated as:

6 $$\widehat{\mathbf{b}}\left(EN\right)=\mathrm{arg}\underset{\mathbf{b}}{\mathrm{min}}\left\{{\left(\mathbf{y}-\mu \mathbf{1}-\mathbf{X}\mathbf{b}\right)}^{\mathrm{^{\prime}}}\left(\mathbf{y}-\mu \mathbf{1}-\mathbf{X}\mathbf{b}\right)+\lambda \sum_{i=1}^{m}\left[\left(1-\alpha \right){b}_{i}^{2}+\alpha |{b}_{i}|\right]\right\},$$

where the value of the second parameter, $\alpha$, is also determined via cross-validation.

The Wald test was used to test if estimates of ${b}_{i}$ from the four models were significantly different from zero. Under the null hypothesis that ${b}_{i}$ = 0, the Wald test statistic is:

7 $${W}_{i}=\frac{{b}_{i}^{2}}{var({b}_{i})},$$

and follows approximately a Chi-square distribution with one degree of freedom.

Association analysis using LMM was performed with the GEMMA package [37]. Since LMM tests one gene at a time, the *p*-value threshold for statistical significance was determined using the Bonferroni correction method, i.e., 0.05/15,092, where 15,092 is the total number of genes identified. The GLMNET/R package was employed for RR, EN, and LASSO computation. In addition, we compared two commonly used methods to determine the tuning parameters ($\lambda$ and $\alpha$) for these three models. One was minimizing cross-validated mean squared prediction error [38]. The corresponding models are referenced as LASSO-Min, EN-Min, and RR-Min. The other was the one standard error rule [39], which uses the tuning parameter values, resulting in errors that are not more than one standard error of the mean cross-validated error above the minimum. The corresponding models are referred to as LASSO-1SE, EN-1SE, and RR-1SE [40]. Since the GLMNET/R package does not provide $var({b}_{i})$, we calculated the empirical error variance using the bootstrap method [41] for the Wald test in the association analyses using RR-Min and RR-1SE. The sample size was 200 and the number of bootstrap replications was 1000. Since RR tests all the genes simultaneously, no multiple-test correction is needed and *p* = 0.05 was used as the threshold. Moreover, LASSO and EN automatically perform variable selection. We compared two strategies to declare candidate genes in the analyses using LASSO and EN, i.e., simply by selecting non-zero effect genes as candidate genes versus filtering genes using the Wald test, as for RR [42].

### Simulation study

We compared the statistical properties of the models (LMM, LASSO-Min, EN-Min, RR-Min, LASSO-1SE, EN-1SE, and RR-1SE) by simulation experiments, using the data for breast muscle weight. In the simulation study, genes were classified into two categories, i.e., trait-relevant genes and trait-neutral genes, based on the analysis of breast muscle weight using EN-Min. Two genes were randomly selected from the category of trait-neutral genes and a non-zero effect of the gene on breast muscle weight was placed on the first gene. For the second gene, its expression level was shuffled by randomly assigning different expression levels to each individual from among the set of actual observed expressions of this gene. The pseudo-phenotype for breast muscle weight was the sum of the original phenotypic value and the effect of the manipulated gene. We examined 23 scenarios, in which we set the proportion of phenotypic variance for breast muscle weight that was contributed by the expression of the selected gene (first gene) equal to 0.01, 0.02, 0.03, 0.04, 0.05, 0.06, 0.08, 0.10, 0.12, 0.14, 0.16, 0.18, 0.20, 0.22, 0.24, 0.26, 0.28, 0.30, 0.34, 0.38, 0.42, 0.46, and 0.50. For each scenario, the simulation was replicated 100 times and the statistical power and type I error rate were computed. Statistical power was defined as the proportion of selected genes with effects that were successfully detected, whereas the type I error was defined as the proportion of the second selected trait-neutral genes that were incorrectly detected.

## Results

In this study, genome-wide gene expression levels of 398 chickens were measured using RNA-seq to detect candidate genes for breast muscle weight and IMF percentage, relative TG, CHO, and PL content (see Additional file 1: Figure S1). We obtained approximately 7.34 billion clean reads, with an average number of 18.41 million reads for each chicken (see Additional file 2: Table S1). The reads were aligned to the reference genome [GRCg6a (GCA_000002315.5)], with mapping rates ranging from 83.87 to 91.16% (see Additional file 2: Table S1). In total, 15,092 coding genes were detected among all samples and used in subsequent analyses (non-coding genes were excluded).

### Population variations of breast muscle weight and IMF contents and phenotypic correlation

Large variations in the traits analyzed were observed. While the coefficient of variation was high for breast muscle weight (CV = 13.9%), the variability levels were even higher for IMF contents (CV > 35%) (Table 1). According to the hierarchical clustering dendrogram, phenotypes for breast muscle weight were substantially different from IMF content (see Additional file 3: Figure S2). For IMF contents, only CHO content was significantly correlated with breast muscle weight (*r* = 0.10, *p* < 0.05, 95% CI [0.00, 0.20]) (Table 2). Among TG, CHO, and PL, only TG content was significantly correlated with IMF percentage (*r* = 0.21, *p* < 0.05, 95% CI [0.11, 0.31]), which can be attributed to the fact that TG is the main component of IMF. The correlation between TG and CHO content was also high (*r* = 0.49, *p* < 0.05, 95% CI [0.41, 0.57]). The close relationship between TG and CHO was also reflected by the dendrogram (see Additional file 3: Figure S2).

**Table 1:** Descriptive statistics for breast muscle weight and IMF contents

Trait	N	Mean	Standard deviation	Coefficient of variation (%)
BMW (g)	381	83.68	11.66	13.93
IMF (%)	398	1.97	0.76	38.77
TG (mg/g)	398	2.65	1.06	39.92
CHO (mg/g)	398	0.73	0.31	42.94
PL (mg/g)	398	0.22	0.08	37.84

*BMW* breast muscle weight, *IMF* intramuscular fat percentage, *TG* relative triglycerides content, *CHO* relative cholesterol content, *PL* relative phospholipids content

**Table 2:** Correlation between traits analyzed

Trait	BMW (g)	IMF%	TG (mg/g)	CHO (mg/g)	PL (mg/g)
BMW (g)		[0.00, 0.20]	[− 0.04, 0.16]	*[0.00, 0.20]*	[− 0.04, 0.16]
IMF (%)	0.10		*[0.11, 0.31]*	[− 0.07, 0.13]	[− 0.06, 0.14]
TG (mg/g)	0.06	*0.21*		*[0.41, 0.57]*	[− 0.09, 0.12]
CHO (mg/g)	*0.10*^a^	0.03	*0.49*		[− 0.17, 0.03]
PL (mg/g)	0.06	0.04	0.02	− 0.07	

Correlation coefficients are under the diagonal, 95% confidence intervals for correlation coefficient are in upper triangle

*BMW* breast muscle weight, *IMF* intramuscular fat percentage, *TG* relative triglycerides content, *CHO* relative cholesterol content, *PL* relative phospholipids content

^a^Correlation coefficients (and their 95% confidence intervals) significantly different from zero are in italics (*p*-value < 0.05)

### Comparison of association study strategies based on gene expression levels

To decide the optimal strategy for association analyses, we compared the statistical properties of various models through extensive simulations. These included LMM and three regularized methods that use different strategies to determine the values of tuning parameters, i.e., LASSO-Min, LASSO-1SE, RR-Min, RR-1SE, EN-Min, and EN-1SE. Although LASSO and EN implement variable selection automatically, we further explored whether significance testing benefited the association analyses. The conclusions were based on the average of 100 replicates for each simulation scenario.

**Fig. 1:**
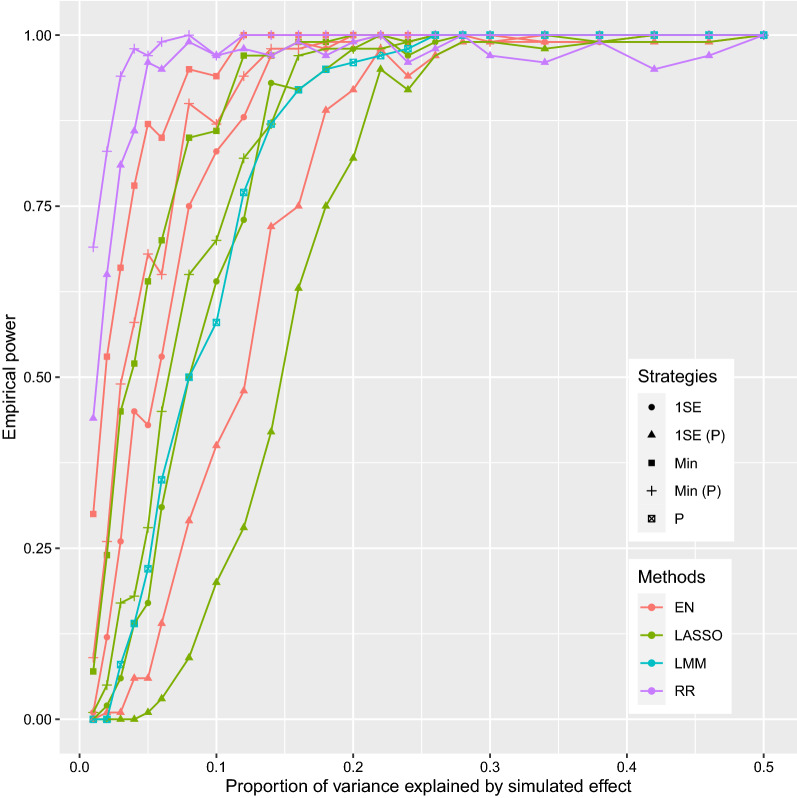
Empirical power of LASSO, EN, RR and LMM based on simulations. X-axis represents the proportion of variance explained by the simulated effect (range from 0.01 to 0.50). 1SE: in this model, the tuning parameters were determined based on the one standard error rule; Min: the tuning parameters were determined by minimizing cross-validated mean squared prediction error; P: the *p*-value threshold was used for statistical significance, which was set at 0.05/15,092 for LMM, and 0.05 for LASSO, EN, and RR. *LASSO* least absolute shrinkage and selection operator, *EN* elastic net, *RR* ridge regression

The results of the simulation study show that detection power increased with size of the simulated effect for all models (Fig. 1). When the level of expression of a gene contributed 50% of the phenotypic variance, all models achieved a statistical power equal to 1. When the level of expression of the gene contributed to a relatively small proportion of the variance (< 0.20), RR achieved the highest power and LASSO achieved the lowest power. For the regularized methods, selecting the model based on the one standard error rule decreased the detection power compared to minimizing the cross-validation error. As anticipated, the significance test reduced the detection power for LASSO and EN models. Moreover, for the variable selection methods LASSO and EN, even with an additional significance test, the models that minimized the cross-validated error achieved greater power than the models that determined parameters based on the one standard error rule and without significance testing. The power of LMM was similar to that of LASSO with parameters determined based on the one standard error rule (LASSO-1SE).

We further compared these models by evaluating their false positive rates. The results showed that RR-min had the highest type I error rates (Table 3). LMM, LASSO-1SE and EN-1SE had the lowest type I error rates, which were exactly zeros. EN-Min with an additional significance test and LASSO-Min exhibited type I error rates less than 0.02. EN-Min without significance testing and RR-1SE exhibited high type I error rates, although lower than RR-Min. Taken together, results of the simulation study demonstrated that the EN-1SE without significance testing was the optimal method for association analyses of expression levels with traits, both in terms of control of type I error rate and detection power.

**Table 3:** Type I error rates of different models in simulations

Heritability	LASSO				EN				RR		LMM
	Min (P)	Min	1SE (P)	1SE	Min (P)	Min	1SE (P)	1SE	Min (P)	1SE (P)	
0.01	0	0	0	0	0	0.02	0	0	0.21	0	0
0.02	0	0	0	0	0.01	0.11	0	0	0.36	0.04	0
0.03	0	0	0	0	0.01	0.11	0	0	0.36	0.04	0
0.04	0	0	0	0	0	0.03	0	0	0.19	0.01	0
0.05	0	0.02	0	0	0.02	0.05	0	0	0.23	0.03	0
0.06	0	0.01	0	0	0.02	0.06	0	0	0.27	0.03	0
0.08	0	0.01	0	0	0.01	0.07	0	0	0.26	0.04	0
0.10	0	0	0	0	0	0.06	0	0	0.31	0.03	0
0.12	0	0	0	0	0	0.04	0	0	0.30	0.03	0
0.14	0.01	0.01	0	0	0.02	0.03	0	0	0.23	0.03	0
0.16	0	0	0	0	0.01	0.02	0	0	0.22	0.01	0
0.18	0	0.01	0	0	0.01	0.04	0	0	0.31	0.05	0
0.20	0	0	0	0	0	0.01	0	0	0.28	0.02	0
0.22	0	0	0	0	0	0.01	0	0	0.24	0.03	0
0.24	0	0.01	0	0	0	0.02	0	0	0.25	0.04	0
0.26	0	0	0	0	0	0	0	0	0.24	0.02	0
0.28	0	0	0	0	0	0.02	0	0	0.20	0.05	0
0.30	0	0	0	0	0	0	0	0	0.25	0.02	0
0.34	0	0.02	0	0	0	0.02	0	0	0.22	0.04	0
0.38	0	0	0	0	0	0	0	0	0.25	0.03	0
0.42	0	0.02	0	0	0	0.02	0	0	0.25	0.06	0
0.46	0	0	0	0	0	0	0	0	0.20	0.03	0
0.50	0	0.02	0	0	0	0.03	0	0	0.32	0.08	0

LASSO: least absolute shrinkage and selection operator; EN: elastic net; RR, ridge regression; LMM: linear mixed model; Min: tuning parameters determined by minimizing cross validated mean squared prediction error; 1SE: tuning parameters in the model determined based on one standard error rule; P: *p*-value threshold were used for statistical significance, which was 0.05/15092 for LMM, and 0.05 for LASSO, EN, and RR

### Identification of candidate genes for breast muscle weight and IMF contents

To improve the overall power and concurrently control the type I error rate in the empirical association analyses, we used three methods that exhibited the lowest type I error rates, i.e., LMM, LASSO-1SE (see Additional file 4: Table S2) and EN-1SE (see Additional file 4: Table S2), to identify candidate genes for the traits analyzed. Significance tests were not performed for LASSO-1SE and EN-1SE (based on the results of the simulation study).

**Fig. 2:**
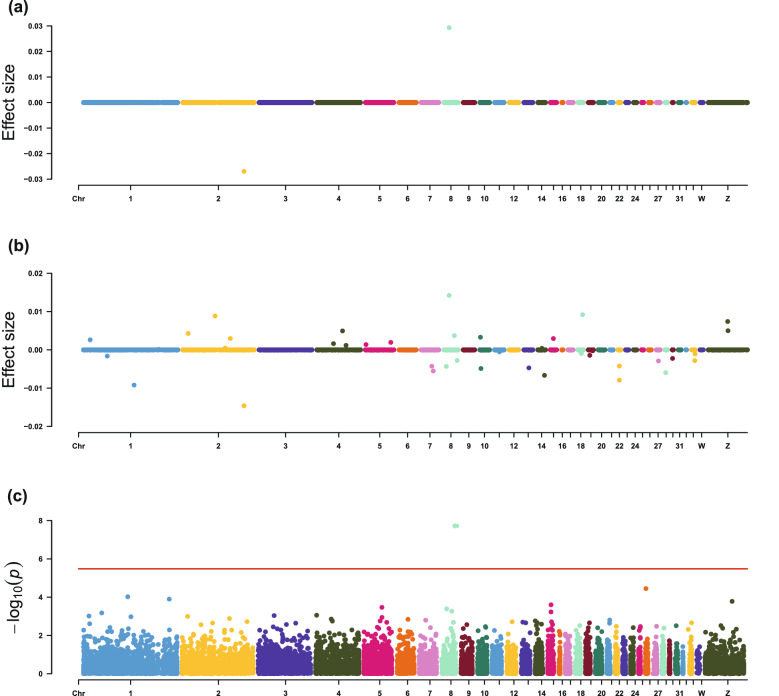
Estimated effect sizes and Manhattan plot of genes based on association analyses for breast muscle weight, using **a** LASSO-1SE, **b** EN-1SE, and **c** LMM. The red line for LMM indicates the thresholds for genome-wide association. *LMM* linear mixed model, *EN-1SE* elastic net with parameters determined based on the one standard error rule, *LASSO-1SE* least absolute shrinkage and selection operator with parameters determined based on the one standard error rule

Using LMM, expression of the *FOXD3* gene was found to be significantly associated with breast muscle weight (Table 4 and Fig. 2). With LASSO-1SE, expressions of three genes had non-zero effects on breast muscle weight, i.e., *PABPC1*, *AMY1A*, and *SERPINB6L*. In accordance with the simulation study, EN-1SE detected more candidate genes than LMM and LASSO-1SE. The expression levels of 43 genes showed non-zero effects on breast muscle weight by EN-1SE (Table 4), including all genes that were identified using LMM and LASSO-1SE.

**Table 4:** Candidate genes for breast muscle weight and IMF contents

Trait	Candidate gene	Position	Method	Effect (SE)^a^	Module
BMW	*PABPC1*	chr2:128,496,930–128,511,621	EN-1SE	− 1.46E−02 (6.90E−03)	Black
			LASSO-1SE	− 2.70E−02 (2.70E−02)	
BMW	*SCAF4*	chr1:106,003,107–106,047,331	EN-1SE	− 9.20E−03 (4.38E−03)	Pink
BMW	*AMY1A*	chr8:11,450,028–11,459,763	EN-1SE	1.42E−02 (7.23E−03)	Black
			LASSO-1SE	2.93E−02 (2.96E−02)	
BMW	*ATG9A*	chr7:22,223,873–22,232,130	EN-1SE	− 4.27E-03 (2.27E−03)	Blue
BMW	*SERPINB6L*	chr2:67,630,807–67,640,267	EN-1SE	8.84E−03 (4.75E−03)	Pink
			LASSO-1SE	7.09E−06 (1.75E−02)	
BMW	*RNPS1*	chr14:14,178,476–14,188,562	EN-1SE	− 6.66E−03 (4.11E−03)	Pink
BMW	*EDC3*	chr10:2,903,180–2,929,046	EN-1SE	− 4.88E−03 (3.08E−03)	Pink
BMW	*NSD1*	chr13:10,923,976–10,979,820	EN-1SE	− 4.71E-03 (2.99E−03)	Pink
BMW	*EEF2*	chr28:1,553,588–1,561,522	EN-1SE	− 5.95E−03 (3.91E−03)	Blue
BMW	*TBC1D16*	chr18:9,562,960–9,588,413	EN-1SE	9.20E−03 (6.21E−03)	–
BMW	*GOLM1*	chr35:40,775,848–40,812,641	EN-1SE	7.40E−03 (5.65E−03)	–
BMW	*PTPN4*	chr7:25,316,975–25,421,700	EN-1SE	− 5.52E−03 (4.30E−03)	Green
BMW	*FGFR1*	chr22:2,646,704–2,665,806	EN-1SE	− 7.92E−03 (6.30E−03)	–
BMW	*FBP2*	chr35:41,434,016–41,454,580	EN-1SE	4.99E−03 (3.99E−03)	Black
BMW	*CETN1*	chr4: 11,283,946–11,288,350	EN-1SE	4.95E−03 (4.01E−03)	Turquoise
BMW	*SCYL3*	chr8:5,441,379–5,454,438	EN-1SE	− 4.33E−03 (3.52E−03)	Pink
BMW	*TLK2*	chr27:4,774,305–4,818,359	EN-1SE	− 2.89E−03 (2.44E−03)	Pink
BMW	*CMPK1*	chr8:22,291,575–22,304,067	EN-1SE	3.73E−03 (3.20E−03)	Blue
BMW	*EIF4EBP1*	chr22:2,560,354–2,564,665	EN-1SE	− 4.23E−03 (4.10E−03)	Blue
BMW	*PFKP*	chr2:11,439,905–11,481,490	EN-1SE	4.29E−03 (4.77E−03)	Yellow
BMW	*PA2G4*	chr33:7,033,372–7,044,838	EN-1SE	− 2.81E−03 (3.53E−03)	Cyan
BMW	*MECP2*	chr30:360,508–372,669	EN-1SE	− 2.24E−03 (2.83E−03)	Blue
BMW	*EP300*	chr1:49,778,616–49,836,401	EN-1SE	− 1.63E−03 (2.19E−03)	Pink
BMW	*LOC107052698*	chr2:99,744,162–99,754,138	EN-1SE	2.98E−03 (4.37E−03)	–
BMW	*TCTN1*	chr15:6,275,491–6,287,845	EN-1SE	2.95E−03 (4.44E−03)	Pink
BMW	*FOXD3*	chr8:28,132,659–28,134,130	EN-1SE	− 2.78E−03 (4.38E−03)	Blue
			LMM	− 3.86E−01 (6.73E−03) (*p* = 1.90E−08)	
BMW	*SRPK2*	chr1:13,957,491–14,095,954	EN-1SE	2.62E−03 (4.35E−03)	–
BMW	*HNRNPA1*	chr33:7,724,007–7,730,306	EN-1SE	− 1.07E-03 (2.24E−03)	Blue
BMW	*AP3B2*	chr10:1,820,119–1,831,108	EN-1SE	3.30E−03 (7.23E−03)	–
BMW	*GTF2IRD1*	chr19:2,866,852–2,916,929	EN-1SE	− 1.43E−03 (3.41E−03)	Blue
BMW	*AKAP5*	chr5:53,099,294–53,106,255	EN-1SE	1.95E−03 (4.84E−03)	Black
BMW	*PURG*	chr4:34,642,974–34,668,274	EN-1SE	1.62E−03 (4.22E−03)	–
BMW	*C5H15orf57*	chr5:1,125,535–1,132,713	EN-1SE	1.38E−03 (4.10E−03)	–
BMW	*BDH2*	chr4:60,973,522–60,987,649	EN-1SE	1.17E−03 (3.85E−03)	Pink
BMW	*CEBPG*	chr11:10,311,180–10,315,686	EN-1SE	− 5.22E−04 (2.14E−03)	Brown
BMW	*HELZ*	chr18:7,145,227–7,219,254	EN-1SE	− 9.49E−04 (4.09E−03)	Blue
BMW	*UBE2O*	chr18:4,379,218–4,428,651	EN-1SE	− 2.71E−04 (1.40E−03)	Pink
BMW	*COQ7*	chr14:8,869,571–8,882,281	EN-1SE	3.96E−04 (2.39E−03)	Black
BMW	*MSANTD3*	chr2:89,075,200–89,090,503	EN-1SE	4.85E−04 (4.01E−03)	Midnightblue
BMW	*VEZF1*	chr19:8,866,657–8,883,692	EN-1SE	− 2.47E−04 (2.49E−03)	Blue
BMW	*UBP1*	chr2:44,569,864–44,605,832	EN-1SE	− 1.48E−04 (2.29E−03)	Pink
BMW	*MZT1*	chr1:157,641,451–157,646,198	EN-1SE	1.25E−04 (2.24E−03)	Turquoise
BMW	*RBL2*	chr11:4,992,998–5,010,973	EN-1SE	− 3.24E−05 (2.34E−03)	Pink
IMF	*THRA*	chr27:7,152,162- 7,163,448	EN-1SE	− 1.04E−16 (9.23E−03)	MEBrown
			LMM	− 2.77E−01 (5.30E−02) (*p* = 2.89E−07)	
TG	*COMMD4*	chr10:3,091,477–3,093,335	LMM	2.84E−01 (5.97E−02) (*p* = 2.87E−06)	MEBrown
TG	*HIST1H110*	chr1:48,103,557–48,104,467	LMM	2.31E−01 (4.89E−02) (*p* = 3.22E−06)	–
CHO	*PEA15L1*	chr23:6,137,230–6,144,560	EN-1SE	1.17E−17 (3.51E−03)	MEGreen
PL	*SCFD1*	chr5:34,370,974–34,415,640	EN-1SE	1.06E−16 (1.21E−02)	MEYellow

*BMW* breast muscle weight, *IMF* Intramuscular fat percentage, *TG* relative triglycerides content, *CHO* relative cholesterol content, *PL* relative phospholipids content, *LMM* linear mixed model, *EN-1SE* elastic net with parameters determined based on one standard error rule, *LASSO-1SE* least absolute shrinkage and selection operator with parameters determined based on one standard error rule

^a^Estimated effects and SE (standard errors) are in standard deviation units

**Fig. 3:**
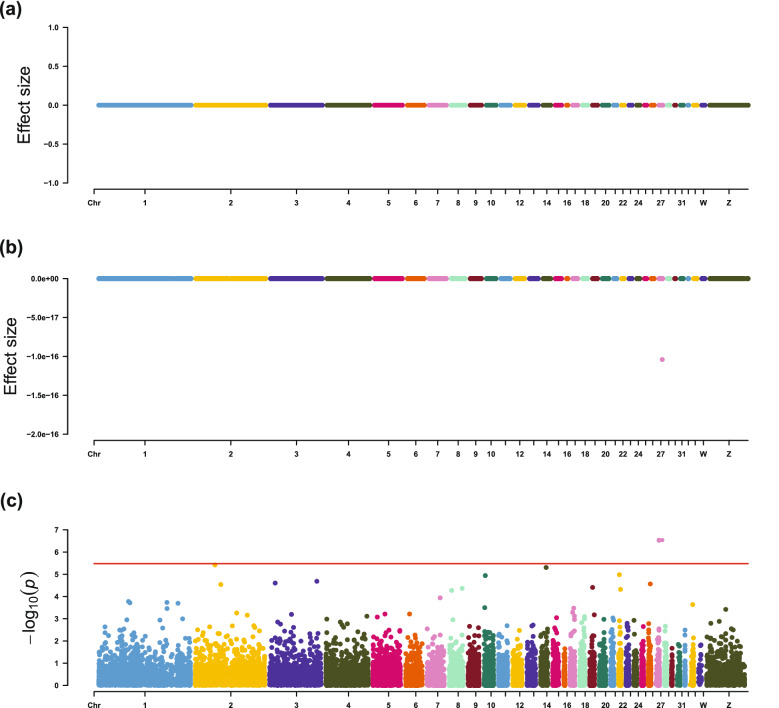
Estimated effect sizes and Manhattan plot of genes based on association analyses for IMF percentage, using **a** LASSO-1SE, **b** EN-1SE, and **c** LMM. The red line for LMM indicates the thresholds for genome-wide association. *LMM* linear mixed model, *EN-1SE* elastic net with parameters determined based on the one standard error rule, *LASSO-1SE* least absolute shrinkage and selection operator with parameters determined based on the one standard error rule

**Fig. 4:**
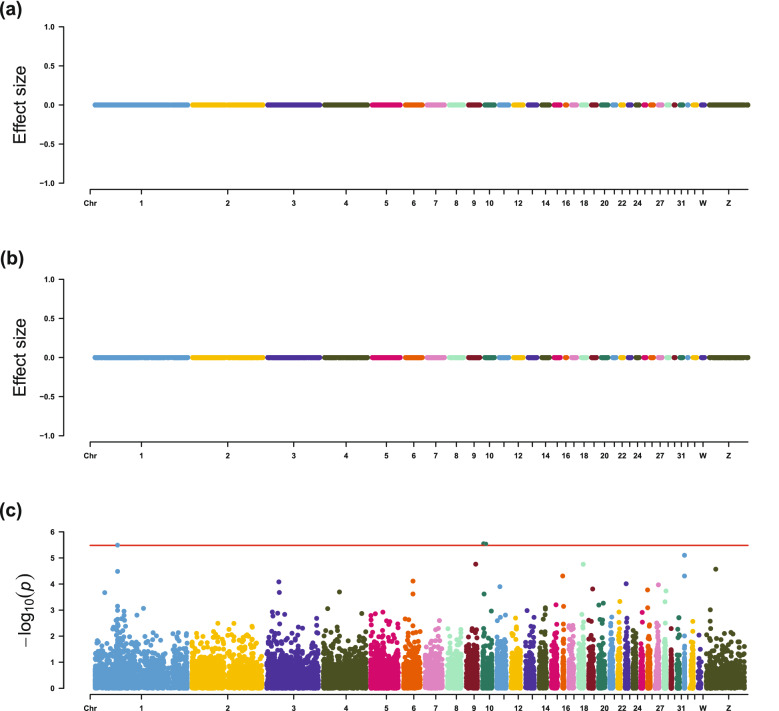
Estimated effect sizes and Manhattan plot of genes based on association analyses for relative TG content, using **a** LASSO-1SE, **b** EN-1SE, and **c** LMM. The red line for LMM indicates the thresholds for genome-wide association. *LMM* linear mixed model, *EN-1SE* elastic net with parameters determined based on the one standard error rule, *LASSO-1SE* least absolute shrinkage and selection operator with parameters determined based on the one standard error rule

**Fig. 5:**
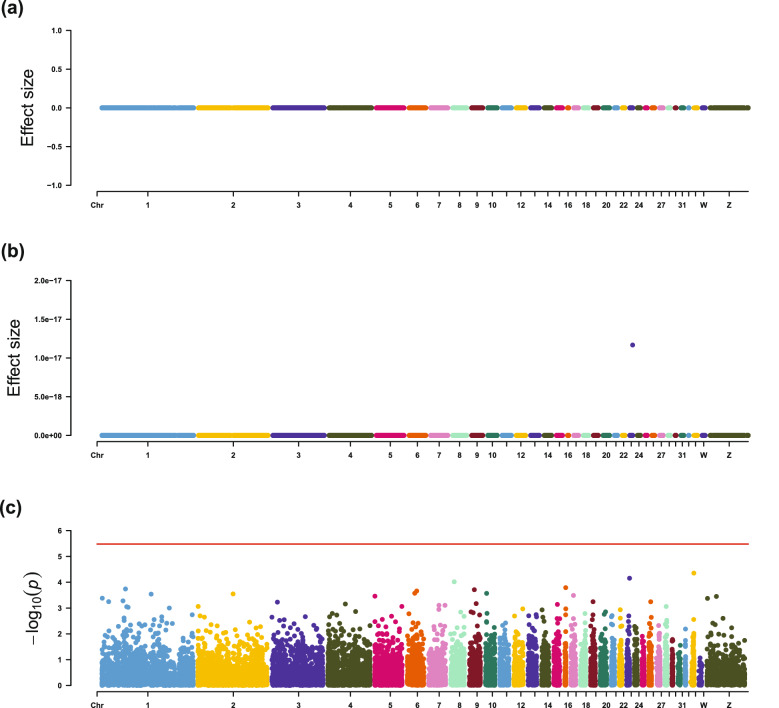
Estimated effect sizes and Manhattan plot of genes based on association analyses for relative CHO content, using **a** LASSO-1SE, **b** EN-1SE, and **c** LMM. The red line for LMM indicates the thresholds for genome-wide association. *LMM* linear mixed model, *EN-1SE* elastic net with parameters determined based on the one standard error rule, *LASSO-1SE* least absolute shrinkage and selection operator with parameters determined based on the one standard error rule

**Fig. 6:**
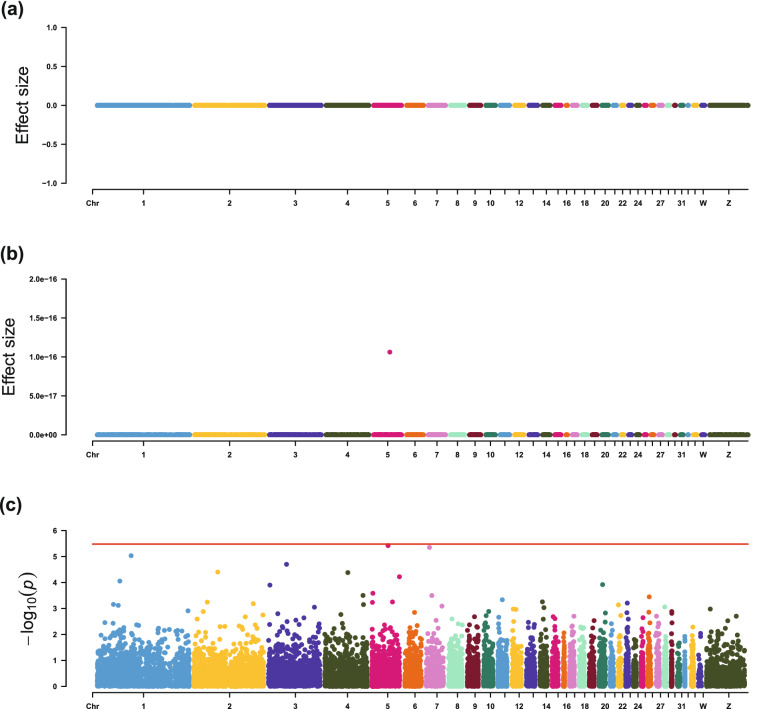
Estimated effect sizes and Manhattan plot of genes based on association analyses for relative PL content, using **a** LASSO-1SE, **b** EN-1SE, and **c** LMM. The red line for LMM indicates the thresholds for genome-wide association. *LMM* linear mixed model, *EN-1SE* elastic net with parameters determined based on the one standard error rule, *LASSO-1SE* least absolute shrinkage and selection operator with parameters determined based on the one standard error rule

For IMF contents, EN-1SE found that the expression levels of *THRA*, *PEA15L1*, and *SCFD1* were associated with IMF percentage, CHO content, and PL content, respectively (Table 4 and Figs. 3, 4, 5 and 6). The association between expression of *THRA* and IMF percentage was also detected by LMM. Although the associations of the expression level of the *PEA15L1* gene with CHO content and the expression level of the *SCFD1* gene with PL content were not significant using LMM, they had very low *p*-values in the LMM analyses, i.e. 7.13 × 10^–5^ and 3.78 × 10^–6^, respectively. In addition, LMM revealed that the expression levels of the *COMMD4* and *HIST1H110* genes were associated with TG content.

We also checked the quantile–quantile (QQ) plots for the LMM analyses, which compare the distribution of − log(*p*-values) observed in the study with the expected distribution under the null hypothesis (see Additional file 5: Figure S3 and Additional file 6: Figure S4). The QQ plots show that the observed distribution of *p*-values was generally as expected, except for low *p*-values, suggesting that LMM effectively controlled type I error rates.

### Characterization of candidate genes for breast muscle weight

**Fig. 7:**
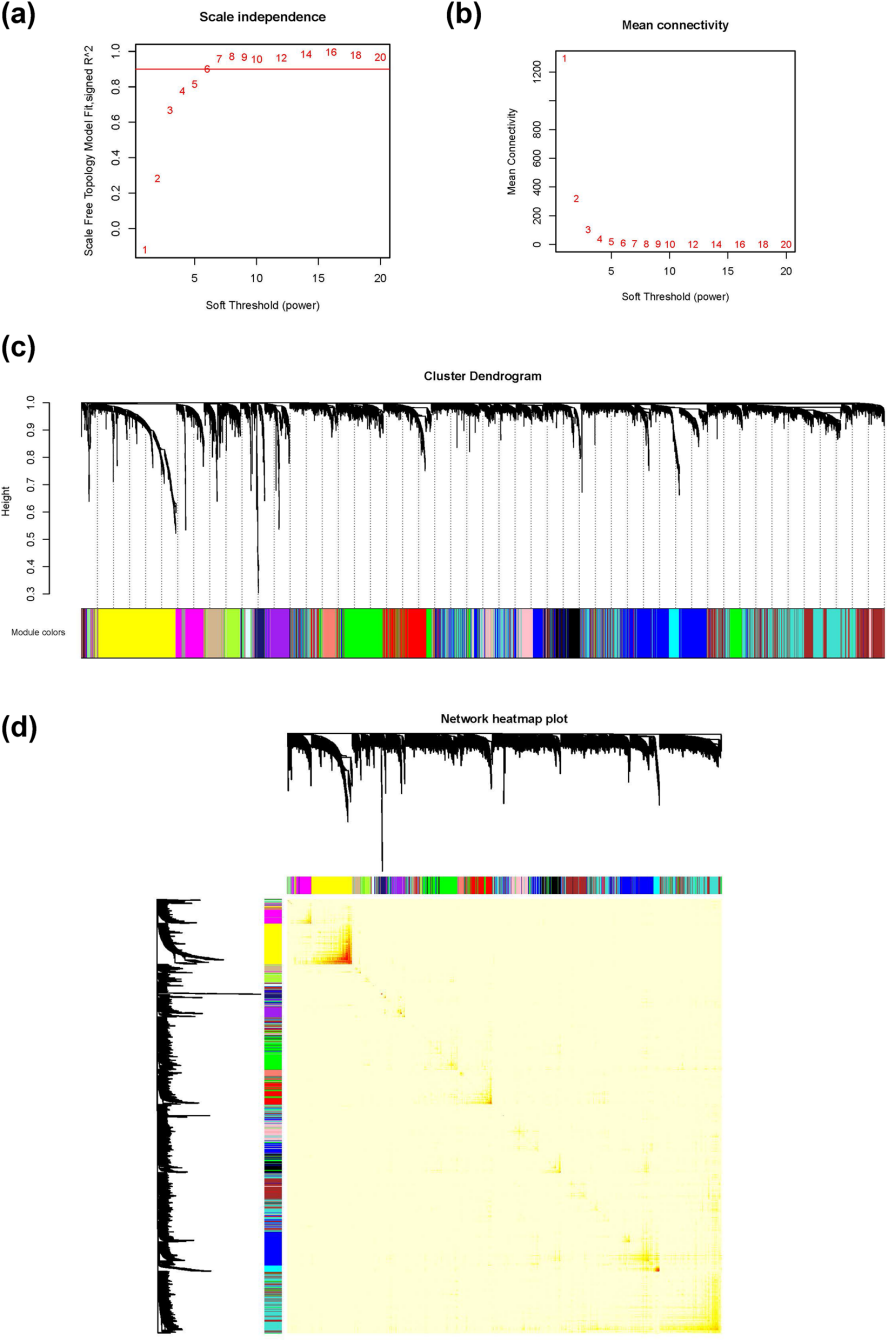
Co-expression analyses of breast muscle weight: **a** analysis of the scale-free fit index for various soft-thresholding powers (*β*), **b** analysis of the mean connectivity for various soft-thresholding powers, **c** clustering dendrogram of genes and, **d** network heatmap plot

In WGCNA, the soft-threshold process transforms the pairwise correlation to an adjacency matrix that mimics the scale-free topology. The soft-thresholding power is recommended as a noise filtering and is a key parameter for subsequent network construction and identification of modules. To optimize this power, the scale-free topology was estimated with the values of power ranging from 1 to 20. It is important to maximize scale-free topology model fit (*R*^2^) while maintaining a relatively large mean number of connections (mean connectivity). When the scale-free topology model fit threshold was set equal to 0.9, the soft-thresholding power was selected as 7 in WGCNA for breast muscle weight (Fig. 7a, b). Nineteen gene co-expression network modules were identified, which consisted of a median of 259 genes (Fig. 7c and see Additional file 7: Table S3). Gene expression profiles were relatively independent between modules (Fig. 7d). Among the 43 candidate genes for breast muscle weight, 35 were located in nine co-expression network modules (Table 4), among which six were significantly correlated with breast muscle weight (*p* < 0.05) (Table 5). GO and KEGG pathway enrichment analyses were performed to determine the potential functions of the correlated modules (see Additional file 8: Tables S4 and S5). For significantly correlated modules (*r* > 0.10, *p* < 0.05) that included detected candidate genes, the MCC score was calculated for each gene and the candidate genes that ranked in the top 10% were recognized as hub genes (see Additional file 9: Table S6).

Thirteen candidate genes were located in the module ‘pink’, which was significantly and negatively correlated with breast muscle weight (*r* = − 0.29, *p* < 0.05). Among the 13 genes, the GO terms of the *RNPS1*, *EDC3*, and *UBP1* genes were enriched and were related to regulation of mRNA metabolic processing, ribonucleoprotein granule, and transcription corepressor activity, respectively. The gene *RNPS1* ranked in the top 15% (47/324) in the module ‘pink’ by MCC, which indicates that it has a relatively high connectivity with other genes (see Additional file 9: Table S6). The gene *NSD1* was also identified as a hub gene and several *NSD1* GO terms were over-represented among the genes in the module ‘pink’, including covalent chromatin modification, peptidyl-lysine modification, nuclear hormone receptor binding, and transcription corepressor activity. The thyroid hormone signaling pathway was also over-represented in the module ‘pink’.

The module ‘black’ included the candidate genes *AKAP5*, *AMY1A*, *COQ7*, *FBP2*, and *PABPC1* and was significantly and positively correlated with breast muscle weight (*r* = 0.16, *p* < 0.05) (Table 4). For the module ‘black’, the over-represented GO terms and KEGG pathways were related to muscle tissue development and muscle contraction. The GO terms of the *COQ7* gene were also enriched among the genes in module ‘black’ and related to coenzyme metabolic process and mitochondrial inner membrane. The GO terms and KEGG pathways of the *FBP2* gene were also over-represented among the genes in module ‘black’, including those related to glucose metabolic processing and contractile fiber.

The candidate genes *CETN1* and *MZT1* were included in the module ‘turquoise’, which was significantly associated with breast muscle weight (*r* = 0.14, *p* < 0.05). The inositol phosphate metabolism KEGG pathway was enriched in the module ‘turquoise’, which was associated with muscle contraction. In the module ‘turquoise’, GO terms relating to protein degradation such as autophagy and proteasomal protein catabolic processing were also enriched. Finally, the spindle GO term of the candidate genes *CETN1* and *MZT1* was enriched among the genes in the module ‘turquoise’.

The candidate gene *PA2G4* was located in the module ‘cyan’ (*r* = − 0.11, *p* < 0.05), for which the ribosome KEGG pathway and GO terms related to both cytoplasmic and mitochondrial ribosome, translation, and focal adhesion were over-represented. In the module ‘cyan’, the functions of the gene *PA2G4* were also enriched, including rRNA metabolic processing, ncRNA processing, ribosome biogenesis, and regulation of translation.

**Table 5:** Correlations between gene co-expression network modules and breast muscle weight

Module	*r*	*p-*value	Module	*r*	*p*-value
*Black*^a^	*0.16*	*2E−03*	Magenta	0.06	2E−01
Blue	− 0.09	1E−01	*Midnight blue*	*0.12*	*2E−02*
*Brown*	− *0.11*	*3E−02*	*Pink*	− *0.29*	*7E−09*
*Cyan*	− *0.11*	*3E−02*	Purple	0.00	1E+00
Green	− 0.02	8E−01	Red	− 0.06	3E−01
Greenyellow	0.01	9E−01	*Salmon*	− *0.15*	*4E−03*
*Grey*	− *0.27*	*9E−08*	Tan	− 0.03	6E−01
Grey60	0.03	6E−01	*Turquoise*	*0.14*	*5E−03*
Lightcyan	0.02	7E−01	Yellow	0.05	3E−01
Lightgreen	0.01	9E−01			

*r*: correlation coefficient

^a^Modules with correlation *p*-values < 0.05 are in italics

The candidate gene *MSANTD3* was included in the module ‘midnight blue’ (*r* = 0.12, *p* < 0.05), in which the KEGG pathways related to cardiomyopathy, and the GO terms related to muscle such as muscle tissue development and muscle contraction, were over-represented. The candidate gene *CEBPG* was included in the module ‘brown’ (*r* = − 0.11, *p* < 0.05), which showed GO term and KEGG pathway enrichment for protein catabolic processing.

### Characterization of candidate genes for IMF contents

**Fig. 8:**
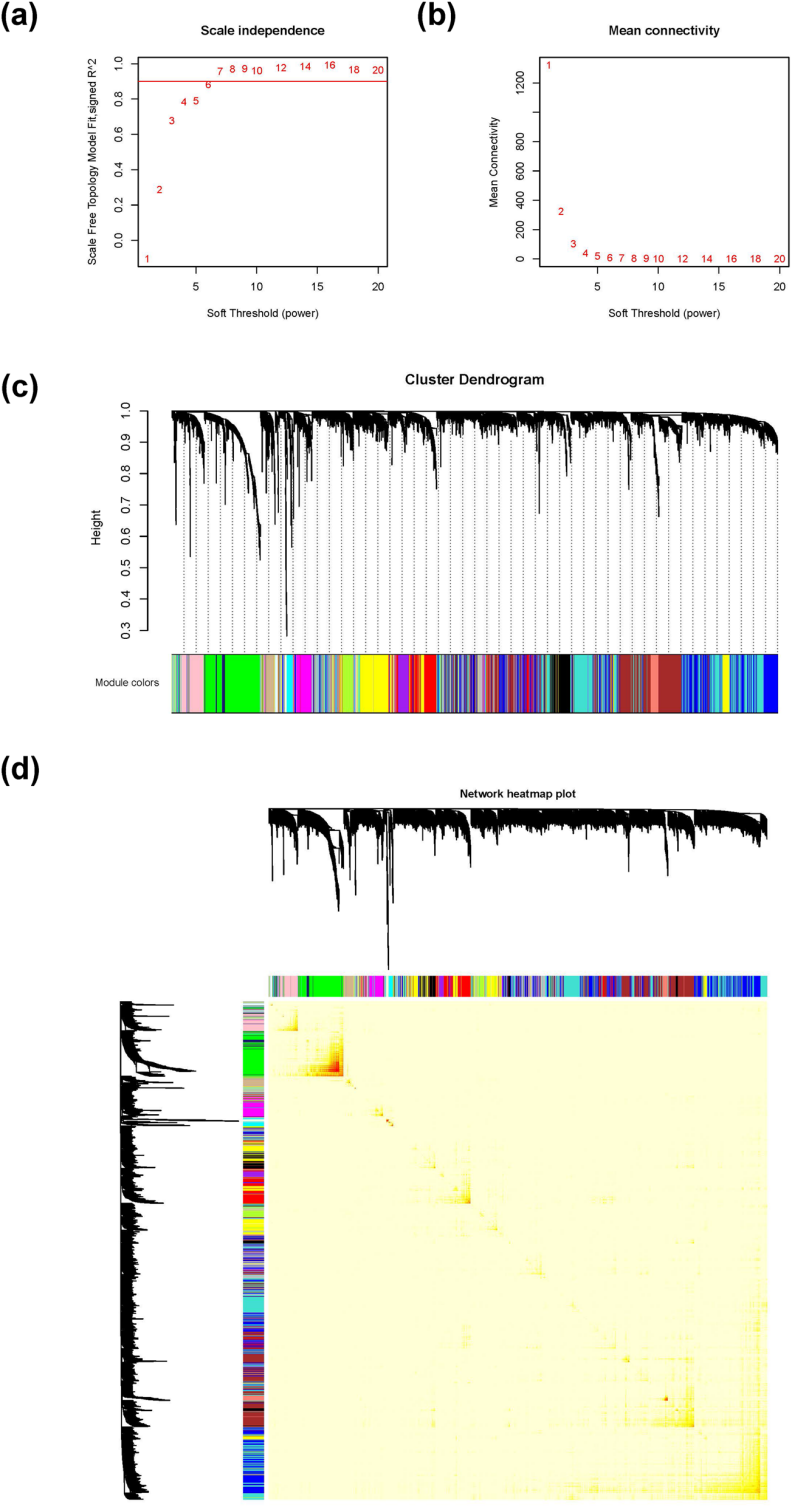
Co-expression analyses of IMF contents: **a** analysis of the scale-free fit index for various soft-thresholding powers (*β*), **b** analysis of the mean connectivity for various soft-thresholding powers, **c** clustering dendrogram of genes, and **d** network heatmap plot

We also set the correlation coefficient threshold equal to 0.9 and selected 7 as the soft-thresholding power for WGCNA analyses of IMF contents (Fig. 8a, b). Twenty network modules were identified by WGCNA, which consisted of a median of 238 genes (Fig. 8c and see Additional file 10: Table S7). Figure 8d shows that gene expression profiles were relatively independent among these modules. The correlation coefficients of gene expression modules with IMF contents are in Table 6.

**Table 6:** Correlations between gene co-expression network modules and IMF contents

Module	IMF percentage		TG content		CHO content		PL content	
	*r*	*p*-value	*r*	*p*-value	*r*	*p*-value	*r*	*p*-value
MEblack	0.00	9E−01	0.02	7E−01	0.05	3E−01	0.02	7E−01
MEblue	0.09	7E−02	− 0.03	6E−01	0.02	4E−01	− 0.06	1E−01
MEbrown	− 0.08	1E−01	− 0.03	6E−01	− 0.03	7E−01	0.06	2E−01
MEcyan	0.05	3E−01	0.03	4E−01	− 0.04	6E−01	0.08	8E−01
*MEgreen*	0.00	1E+00	− 0.02	9E-−01	− 0.09	5E−01	− *0.09*	*2E*−*02*
*MEgreenyellow*	0.03	5E−01	− 0.06	7E−01	− 0.01	1E−01	− *0.03*	*8E*−*03*
*MEgrey*^a^	− *0.20*	*7E*−*05*	0.04	4E−01	0.02	4E−01	*0.01*	*9E*−*06*
MEgrey60	0.01	9E−01	0.09	8E−01	0.05	5E−01	− 0.01	7E−01
MElightcyan	0.06	2E−01	0.04	2E−01	0.06	5E−01	0.01	2E−01
MElightgreen	− 0.02	7E−01	0.00	4E−01	0.03	3E−01	0.11	9E−01
MElightyellow	0.01	8E−01	0.02	2E−01	0.03	9E−01	− 0.01	6E−01
*MEmagenta*	*0.12*	*2E*−*02*	0.01	7E−02	0.03	3E−01	0.02	8E−01
MEmidnightblue	0.03	6E−01	0.00	6E−01	0.09	5E−01	0.07	9E−01
MEpink	− 0.01	9E−01	0.02	1E+00	− 0.04	9E−02	0.01	2E−01
MEpurple	0.03	5E−01	− 0.03	5E−01	− 0.02	7E−01	0.04	5E−01
MEred	0.09	8E−02	0.00	1E+00	− 0.01	9E−01	0.06	3E−01
MEsalmon	− 0.02	7E−01	0.06	6E−01	0.04	5E−01	− 0.06	2E−01
MEtan	− 0.03	6E−01	0.02	5E−01	− 0.08	6E−01	− *0.13*	*3E*−*02*
MEturquoise	− 0.06	2E−01	− 0.03	7E−01	− 0.03	8E−02	− 0.11	6E−02
*MEyellow*	*0.15*	*2E*−*03*	− 0.04	6E−01	− 0.04	4E−01	− 0.22	8E−01

*r*: correlation coefficient

^a^Modules with correlation *p*-values < 0.05 are in italics

The candidate gene *SCFD1* for PL content was located in the module ‘MEyellow’, which was significantly and positively correlated with IMF percentage (*r* = 0.15, *p* < 0.05). The GO terms of the *SCFD1* gene, i.e., autophagy and vacuole organization, were enriched in this module. Moreover, GO terms of membrane coating and regulation of lipid metabolic processing were enriched in the ‘MEyellow’ module (see Additional file 11: Table S8). The KEGG pathways of the FoxO signaling pathway, the AMPK signaling pathway, insulin resistance, and autophagy were also over-represented in the ‘MEyellow’ module (see Additional file 12: Table S9).

The candidate gene *PEA15L1* for CHO content was included in the ‘MEgreen’ module, which was significantly and positively correlated with PL content (*r* = 0.11, *p* < 0.05). Many GO terms were enriched in the ‘MEgreen’ module, including those related to differentiation, proliferation, and activation of leukocyte, coagulation, and membrane, etc. In the ‘MEgreen’ module, pathway of leukocyte transendothelial migration was also over-represented.

## Discussion

In the recent decades, the demand for poultry has continuously increased. For broiler chicken production, both breast muscle weight and IMF content are important economic traits. While linkage analyses and GWAS have successfully identified many relevant QTL in broiler chickens, the functional significance of these associations remains elusive due to the inability to accurately map them to tissue-specific and tissue-relevant genes. In recent years, some studies have performed differential gene expression analyses to identify candidate genes for breast muscle weight and IMF content [7, 15–18]. However, the sample sizes in these previous studies were relatively small. Considering that sample size is an important factor that determines the power of detection, we used transcriptome sequences of approximately 400 chickens to detect candidate genes for breast muscle weight and IMF content (IMF percentage, TG content, CHO content, and PL content). Based on the results of extensive simulation analyses, optimized association analyses were conducted, and candidate genes were identified for breast muscle weight (43 genes), IMF percentage (1), TG content (2), CHO content (1), and PL content (1). Many of the identified genes were previously demonstrated to have effects on the corresponding traits.

Breast muscle weight and IMF content are complex traits and their genetic bases have not yet been comprehensively understood. Similar to the results in our study, Liu et al. [8] detected fewer associated SNPs for IMF in breast than for breast muscle weight. More specifically, they detected 19 SNPs that were significantly associated with breast muscle weight, of which 12 were significant at the genome level. For IMF, only two SNPs with suggestive significance were detected. Therefore, the smaller number of candidate genes for IMF contents found in our study may be due to the more complex genetic basis or the smaller proportion of variance explained by individual genes for IMF content.

None of the associations between candidate genes and traits found in our study were catalogued for chicken in the animal QTLdb (accessed 21 Feb 2021) [14]. This could be because candidate genes were identified based on the association of their level with traits, while the candidate genes listed in the animal QTLdb are associated with traits at the DNA level.

### Performance of statistical methods used in the association analyses

In association analyses based on gene expression levels, we compared commonly used GWAS methods, including LMM, LASSO-Min, LASSO-1SE, EN-Min, EN-1SE, RR-Min, and RR-1SE. First, we evaluated the performance of these methods through extensive simulations. As indicated by the results of WGCNA, expression levels of genes are correlated with each other. The RR method shrinks the coefficients of correlated predictors equally towards zero. In contrast, the LASSO method keeps only the strongest predictor among the correlated group of genes [34]. The EN method is a compromise between the RR and LASSO methods and results in a grouping effect that keeps strongly correlated predictors together in the model [36]. Our results showed that the EN method generally resulted in higher power than the LMM and LASSO methods, and in lower type I error rates compared to RR. These results are consistent with the expected outcomes for these methods and with outcomes reported by other studies that tested these methods [43]. In our study, power of the LMM method was similar to that of the LASSO method, and lower than that of the EN method. This may be because the polygenic effect that comprises the effects of all genes absorbed the signal of the tested gene in the analysis using LMM and, thus, reduced power [44].

For the three regularized linear regression methods, using tuning parameters that minimized the mean squared prediction error (LASSO-Min, EN-Min, RR-Min) resulted in both higher power and higher type I error rates compared to using tuning parameters that were determined based on the one standard error rule (LASSO-1SE, EN-1SE, RR-1SE). These results are consistent with those from a previous GWAS [40]. Moreover, the one standard error rule has been favored because it acknowledges the fact that the tradeoff curve is estimated with error and hence takes a conservative approach [39].

The LASSO and EN regularized linear regression models implicitly performed variable selection. We investigated if it is necessary to also test the significance of predictors with non-zero effects for these methods, as in Wei et al. [42]. The results show that, for LASSO-Min and EN-Min, also selecting variables based on a Wald test decreased the type I error rate. However, both LASSO-1SE and EN-1SE effectively controlled the type I error rates, and so conducting the additional step of the Wald test decreased their power of detection. Thus, for association analyses using LASSO-1SE and EN-1SE, genes with non-zero effect estimates can be directly recognized as candidate genes.

LASSO, EN, and RR shrink the coefficients towards zero, i.e., they introduce bias in the estimates. For example, in our simulation study, when the gene effect was set to 0.50 standard deviation units, corresponding to 20% of the variance, the average estimate using EN-1SE was 0.18 ± 0.08 (100 replicates) standard deviation units. As a result, the phenotypic variances explained by candidate genes could not be accurately estimated in the empirical analyses. In addition to identifying candidate genes, we were also interested in determining the direction of their effects. For a candidate gene with an effect that is significantly different from zero, we could determine the direction of its effect based on the sign of the estimated effect, with a certain low error rate. In addition, biological functions of well-studied genes could be used to assist in determining the directions of the effects. For example, the sign of the estimated effect of the *THRA* gene on IMF percentage was negative (Table 4). In addition, *THRA* encodes the receptor for thyroid hormone and is related to energy expenditure. Thus, a higher expression of *THRA* is expected to result in less IMF. Taken together, we could confidently infer that the expression of *THRA* had a negative effect on IMF percentage.

Transcriptome-wide association studies (TWAS) have been widely used to test the association between traits and genetically predicted gene expression levels in humans [45–47]. In these studies, cis-heritable expression levels of genes were computed based on local genotype data. We did not perform a TWAS because the sampled individuals were not genotyped using a SNP chip or whole-genome resequencing. Moreover, a high false positive rate was found for variants discovered by using RNA-seq, which was attributed in part to the effects of RNA editing [48]. When accurate SNPs of individuals are available, TWAS could be performed to detect associations between traits and cis-heritable gene expressions.

### Candidate genes for breast muscle weight were mainly involved in muscle or adipocyte development

Breast meat consists mainly of skeletal muscle and IMF. For breast muscle weight, LMM identified *FOXD3* and LASSO-1SE identified *PABPC1*, *AMY1A*, and *SERPINB6* as candidate genes. These four genes were also identified by EN-1SE. *FOXD3* has been shown to have functions upstream of genes required for skeletal muscle development [49]. The RNA binding protein PABPC1 is known to have an important role in determining protein synthesis rates, and upregulation of *PABPC1* in adult hearts increases heart size and heart-to-body weight ratio [50]. Hence, we speculate that *PABPC1* has a similar positive effect on the development of breast skeletal muscle. *AMY1A* encodes salivary amylase and its copy number is associated with obesity risk [51]. *SERPINB6* encodes a protein of the serpin (serine proteinase inhibitor) superfamily and of the ovalbumin-serpin subfamily. GO annotations related to *SERPINB6* include serine-type endopeptidase inhibitor activity and protease binding. Further work is needed to understand how *SERPINB6* influences traits related to breast muscle.

EN-1SE detected 39 genes for breast muscle weight, in addition to *FOXD3, PABPC1*, *AMY1A*, and *SERPINB6*. Some of these genes were suggested to have effects on breast muscle weight based on previous studies. In general, their functions can be classified into two categories, i.e., genes associated with muscle development, and genes associated with obesity or adipocyte development.

Previous studies reported or indicated that eight genes directly or indirectly regulate muscle development (*TBC1D16*, *FGFR1*, *EIF4EBP1*, *PA2G4*, *HNRNPA1*, *GTF2IRD1*, *VEZF1*, *SRPK2*). The *TBC1D16* gene has been reported to be differentially methylated and differentially expressed in samples of subcutaneous adipose tissue between obese and non-obese human individuals [52, 53]. Jacobsen et al. [54] compared TBC1D16 protein levels in adipocytes of obese and lean pigs and found that the level of the protein translated from its short transcript tended to be higher in obese pigs. The fibroblast growth factor (FGF) signal transduction cascade has been shown to regulate myogenic cell proliferation and differentiation, which is mediated by a fibroblast growth factor receptor (FGFR) [55, 56]. Regulation of *FGFR1* gene expression is known to have a critical role in the development of skeletal muscle [57]. EIF4EBP1, the binding protein and silencer of a key elongation factor in protein synthesis (EIF4), is involved in the mammalian target of rapamycin signaling, which regulates muscle protein synthesis [58, 59]. Moreover, EIF4EBP1 was identified as a key TOR-dependent regulator of muscle fiber size in response to muscle activity [60]. In chickens, the gene was also shown to be differentially expressed in leg muscle at two developmental stages during early growth, suggesting that it has a function in regulating chicken growth and development [61]. PA2G4, also named EBP1, is a ubiquitously expressed DNA and RNA binding protein that regulates embryonic muscle progenitors and adult muscle stem cells. Down-regulation of *PA2G4* has been shown to prohibit myogenic differentiation of muscle progenitors in chick embryos [62]. *HNRNPA1*, which is involved in various cellular functions related to RNA processing, has been reported to have a role in smooth muscle differentiation [63]. Similarly, GTF2IRD1 may be a transcription regulator involved in cell-cycle progression and skeletal muscle differentiation [64]. VEZF1 has recently been identified as a novel transcription factor necessary for *β*-adrenergic stress-induced increases in cardiac growth and contraction [65]. Serine‐arginine protein kinase (SRPK) is well known for its role in regulation of alternative splicing [66]. The chicken genome includes three *SRPK* genes (*SRPK1*, *SRPK2*, *SRPK3*). Expression of *SRPK3* has been reported to promote the splicing of the MEF2Cα2 isoform, which plays an important role in normal myogenesis [67]. Moreover, *SRPK3* has been shown to be differentially expressed and alternatively spliced in four muscle tissues collected from two chicken breeds at different ages, suggesting its function in muscle development [68].

Three candidate genes (*CEBPG*, *UBE2O* and *RBL2*) were identified to be involved in the metabolism of adipocytes. The transcription factor CEBP family is known to have a role in cell proliferation and differentiation of several cell types [69]. Among them, *CEBPG* has been identified as an activator of *TORC2*, which plays a key role in adipogenesis [70]. *UBE2O* has been previously implicated in regulation of adipogenesis in vitro [71]. *RBL2* plays a role in preadipocyte proliferation and differentiation [72] and it has been suggested that a variant in the *FTO* gene is strongly associated with obesity and influences *RBL2* expression, which impacts obesity risk [73].

The candidate genes *RNPS1*, *EDC3*, and *UBP1* were co-expressed in the module ‘pink’ and their common functions related to regulation of transcription and translation were enriched in this module. More specifically, *RNPS1* has been recognized as an activator of pre-mRNA splicing and shown to regulate alternative splicing in vivo [74, 75]. Furthermore, the encoded protein facilitates the 3′ end processing of mRNA and improves translational activity [76, 77]. Based on its MCC value rank in the module ‘pink’, *RNPS1* was also found to have a relatively high connectivity with the other genes (see Additional file 9: Table S6), which suggests that it may exert a regulation function on many genes. The *EDC3* gene affects the decay rates and/or steady-state levels of multiple mRNAs [78, 79] and is thought to play a scaffolding role in the assembly of a larger decapping complex [80]. UBP1, together with TFCP2 and TFCP2L1, constitute a distinct subfamily of grainyhead-like transcription factors [81]. UBP1 has been demonstrated to play a crucial role in regulation of extraembryonic angiogenesis and mice that lack *UBP1* expression exhibited growth retardation at embryonic day 10.5 and died 1 day later [82]. In addition to these three genes, the candidate gene *NSD1* is a critical regulator of transcription through histone modification and chromatin modelling [83]. As the hub gene in the co-expression network module ‘pink’ (see Additional file 9: Table S6), *NSD1* may regulate the transcription of many genes in this module*.* Therefore, *RNPS1*, *EDC3*, *UBP1*, and *NSD1* may indirectly affect muscle or adipocyte development by regulating the transcription and translation of key genes involved in the corresponding biological process, similar to the functions of the candidate genes *HNRNPA1*, *GTF2IRD1* and *VEZF1*.

### Candidate genes for IMF content were marginally related to lipid metabolism

The *thyroid hormone receptor alpha* (*THRA*) gene was found to be associated with IMF percentage by EN-1SE and LMM. Thyroid hormones influence not only skeletal muscle homeostasis and functions but also nearly all the other major metabolic pathways, including synthesis, mobilization and degradation of lipids [84]. The *COMMD4* and *HIST1H110* genes were found to be associated with TG content by LMM. Although the molecular function of *HIST1H110* is poorly studied, it has previously been identified as a differentially expressed gene in chickens with divergent residual feed intakes [85]. The *HIST1H110* gene was also included in two gene sets that were shown in a previous study to interact with other gene sets involved in body weight at birth in chickens [86]. Further study is needed to investigate how *COMMD4* influences lipid metabolism.

The genes *SCFD1* and *PEA15L1* were found to be associated with PL and CHO content, respectively. *SCFD1* was located in the ‘MEyellow’ module, for which the GO term autophagy of *SCFD1* was enriched. Autophagy has previously been demonstrated to have a role in lipid metabolism [87, 88]. Moreover, enriched GO terms in the ‘MEyellow’ module included other terms related to lipid metabolism, including membrane coating and regulation of lipid metabolic processing. The KEGG FoxO [89] and AMPK [90] signaling pathways were also over-represented in the ‘MEyellow’ module and are also involved in lipid metabolism. The *PEA15L1* gene was located in the ‘MEgreen’ module, for which GO terms and KEGG pathways related to immune cells (leukocyte differentiation, proliferation, activation, etc.) were significantly enriched. It has been shown that lipids affect innate and adaptive immune responses, since the alteration of the lipid metabolism affects the activation, differentiation, and function of immune cells [91]. Therefore, the relationship between the ‘MEgreen’ module and lipid content may result from the PL content affecting the expression of genes in this module.

### Evaluating candidate genes identified by gene expression microarrays in other species

De Jager et al. [92] and Guo et al. [93] performed genome-wide gene expression analyses to detect candidate genes for IMF percentage using microarray data in cattle and sheep, respectively. Guo et al. [93] also incorporated the results from De Jager et al. [92] and found that 30 lipid metabolism genes were correlated with IMF percentage in both cattle and sheep. Among these, the orthologs of 22 genes were found for chicken using Biomart in Ensembl (http://www.ensembl.org/biomart/martview). Of these, only *ACER3* ranked in the top 100 genes in our study based on Pearson’s correlations between gene expression and IMF percentage (rank = 58/15092) (see Additional file 13: Table S10). Consistently, *ACER3* had the smallest *p*-value for IMF percentage using LMM (7.66E−3), but it was not significant at the genome level (threshold = 0.05/15092 = 3.31E−6).

Two factors may explain the different results from these two previous studies and from our study. On the one hand, compared with ruminants, it is possible that in chickens, different candidate genes and biological processes are involved in lipid metabolism. On the other hand, the two previous studies used a simple Pearson’s correlation and additional biological information to control high false positive and false negative rates in the detection of candidate genes. Compared to the simple Pearson’s correlation, the LMM used in our study also considered random polygenic effects to correct for population structure. This strategy is similar to that used in GWAS, which could decrease the false positive rate. Moreover, as indicated by our simulation study, LMM could not achieve a power of 1 when the simulated gene explained less than 26% of the phenotypic variance, which is often the case. Hence, it is possible that most of the 22 genes were false negatives in our study. Therefore, the different results may also be due to the use of different statistical methods with different false positive and false negative rates.

## Conclusions

We identified 43 genes for which total expression was associated with breast muscle weight, as well as genes the expression of which was associated with IMF percentage (1), TG content (2), CHO content (1), and PL content (1), making them candidate genes for these respective traits. Additional research is required to validate the associations and to further unravel the molecular mechanisms of the identified candidate genes. These results provide new candidate genes and clues for deciphering the molecular mechanisms that underlie muscle development and lipid deposition of breast muscle in chickens.

## Supplementary Information

**Additional file 1: Figure S1.** Schematic diagram of the study design.

**Additional file 2: Table S1.** Number of clean reads and mapping rate of the 399 samples.

**Additional file 3: Figure S2.** Hierarchical clustering dendrogram of the traits analyzed.

**Additional file 4: Table S2.** Values of tuning parameters used in LASSO-1SE and EN-1SE in association analyses.

**Additional file 5: Figure S3.** Quantile–quantile (QQ) plot of the association analysis for breast muscle weight using a linear mixed model. The grey area in the QQ plot represents the 95% confidence interval around the test statistic.

**Additional file 6: Figure S4.** Quantile–quantile (QQ) plots of the association analyses for (a) IMF percentage, (b) TG content, (c) CHO content, and (d) PL content using linear mixed model. The grey areas in the QQ plots represent the 95% confidence intervals around the test statistics.

**Additional file 7: Table S3.** Genes in each WGCNA module for breast muscle weight.

**Additional file 8: Table S4.** GO terms enriched in the WGCNA modules correlated with breast muscle weight. **Table S5.** KEGG pathway enriched in the WGCNA modules correlated with breast muscle weight.

**Additional file 9: Table S6.** Rank of candidate genes for breast muscle weight within co-expression network modules by MCC.

**Additional file 10: Table S7.** Genes in each WGCNA module for IMF contents.

**Additional file 11: Table S8.** GO terms enriched in the WGCNA modules correlated with IMF contents.

**Additional file 12: Table S9.** KEGG pathway enriched in the WGCNA modules correlated with IMF contents.

**Additional file 13: Table S10.** Results from LMM and Pearson correlation analyses for IMF percentage.

## Data Availability

The datasets analyzed during the current study are available from the corresponding author on reasonable request.
