# Supplementary material for: Large-scale transcriptome sequencing in broiler chickens to identify candidate genes for breast muscle weight and intramuscular fat content
Source: Genet Sel Evol. 2021 Aug 16;53:66. doi: 10.1186/s12711-021-00656-9 (PMC8369645; doi:10.1186/s12711-021-00656-9)
Supplement: Supplementary file 1 — Additional file 1: Figure S1. Schematic diagram of the study design. [file 12711_2021_656_MOESM1_ESM.pdf]

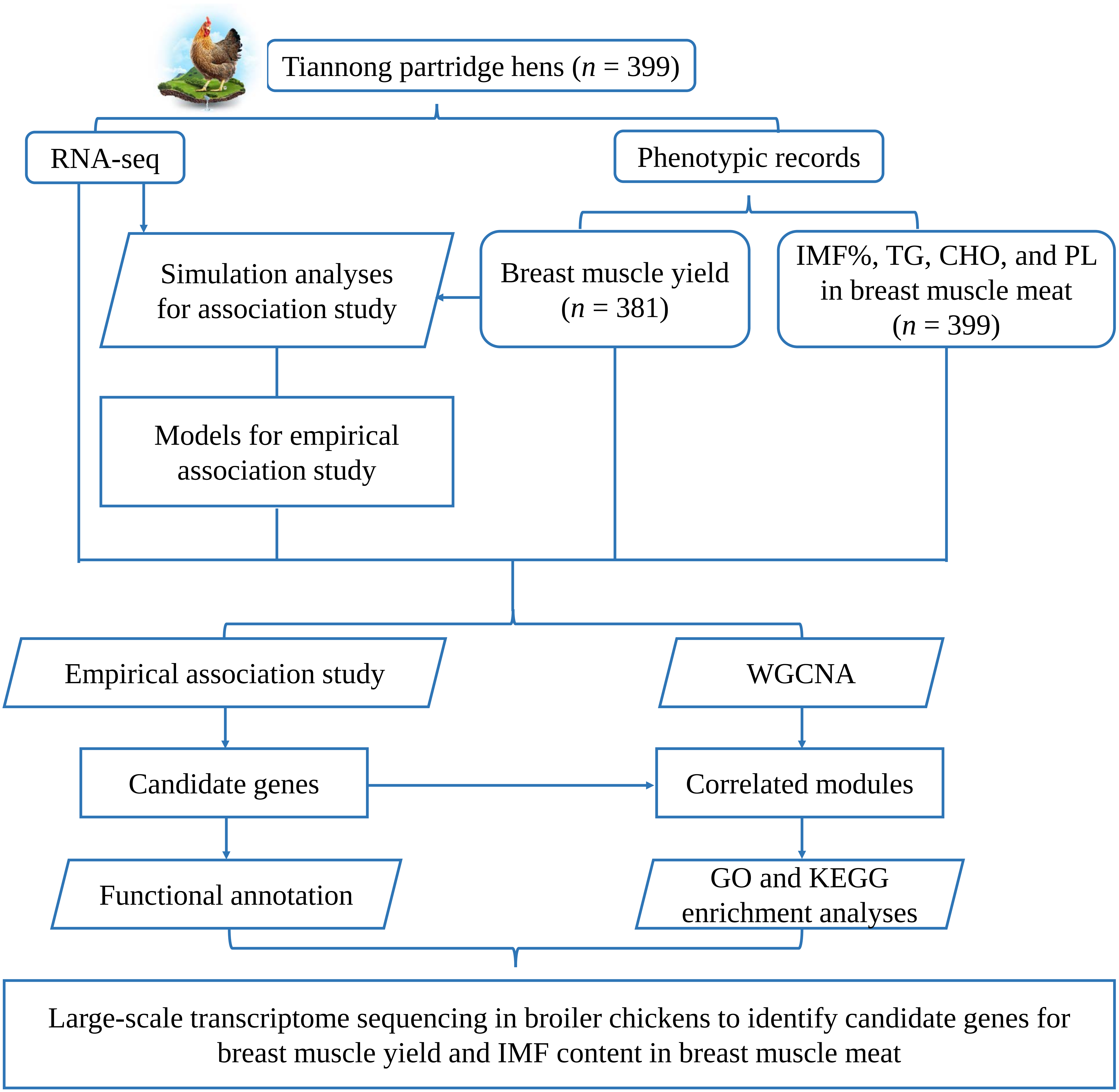
Tiannong partridge hens (n = 399)
RNA-seq
Phenotypic records
Simulation analyses for association study
Breast muscle yield (n = 381)
IMF%, TG, CHO, and PL in breast muscle meat (n = 399)
Models for empirical association study
Empirical association study
WGCNA
Candidate genes
Correlated modules
Functional annotation
GO and KEGG enrichment analyses
Large-scale transcriptome sequencing in broiler chickens to identify candidate genes for breast muscle yield and IMF content in breast muscle meat
