# Supplementary figures and images for: Large-scale transcriptome sequencing in broiler chickens to identify candidate genes for breast muscle weight and intramuscular fat content
Source: Genet Sel Evol. 2021 Aug 16;53:66. doi: 10.1186/s12711-021-00656-9 (PMC8369645; doi:10.1186/s12711-021-00656-9)

Height

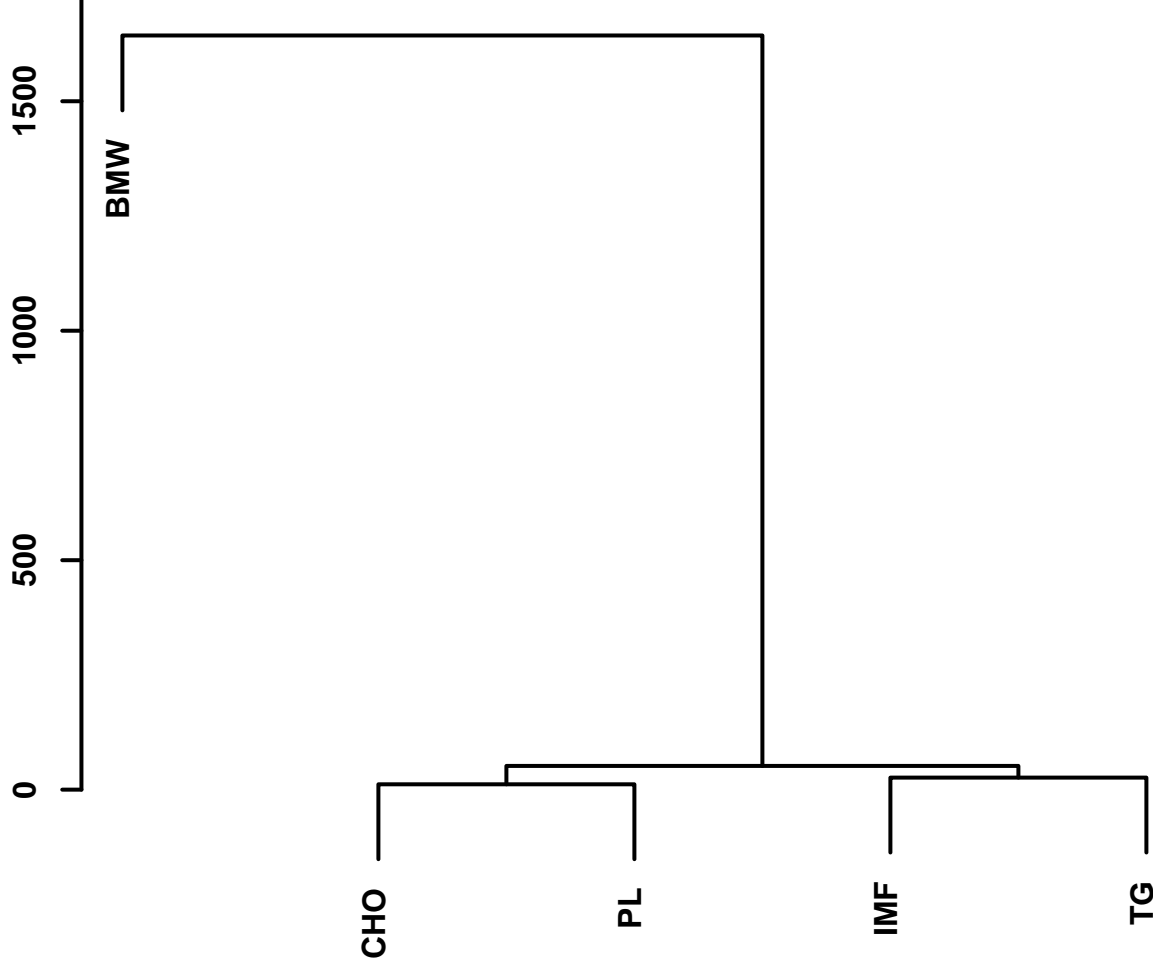

Supplement: Supplementary file 3 — Additional file 3: Figure S2. Hierarchical clustering dendrogram of the traits analyzed. [file 12711_2021_656_MOESM3_ESM.pdf]

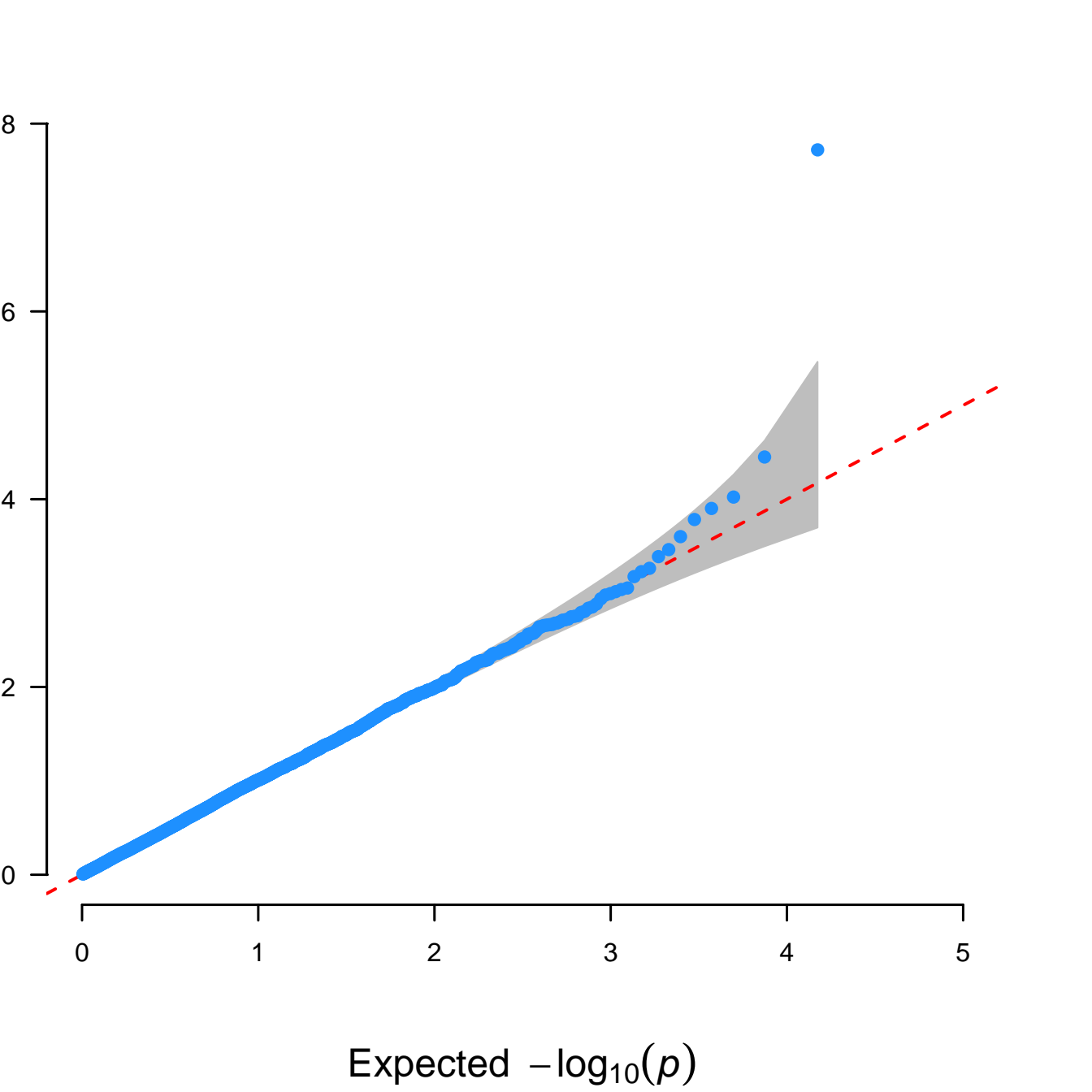

Supplement: Supplementary file 5 — Additional file 5: Figure S3. Quantile–quantile (QQ) plot of the association analysis for breast muscle weight using a linear mixed model. The grey area in the QQ plot represents the 95% confidence interval around the test statistic. [file 12711_2021_656_MOESM5_ESM.pdf]

**(a)**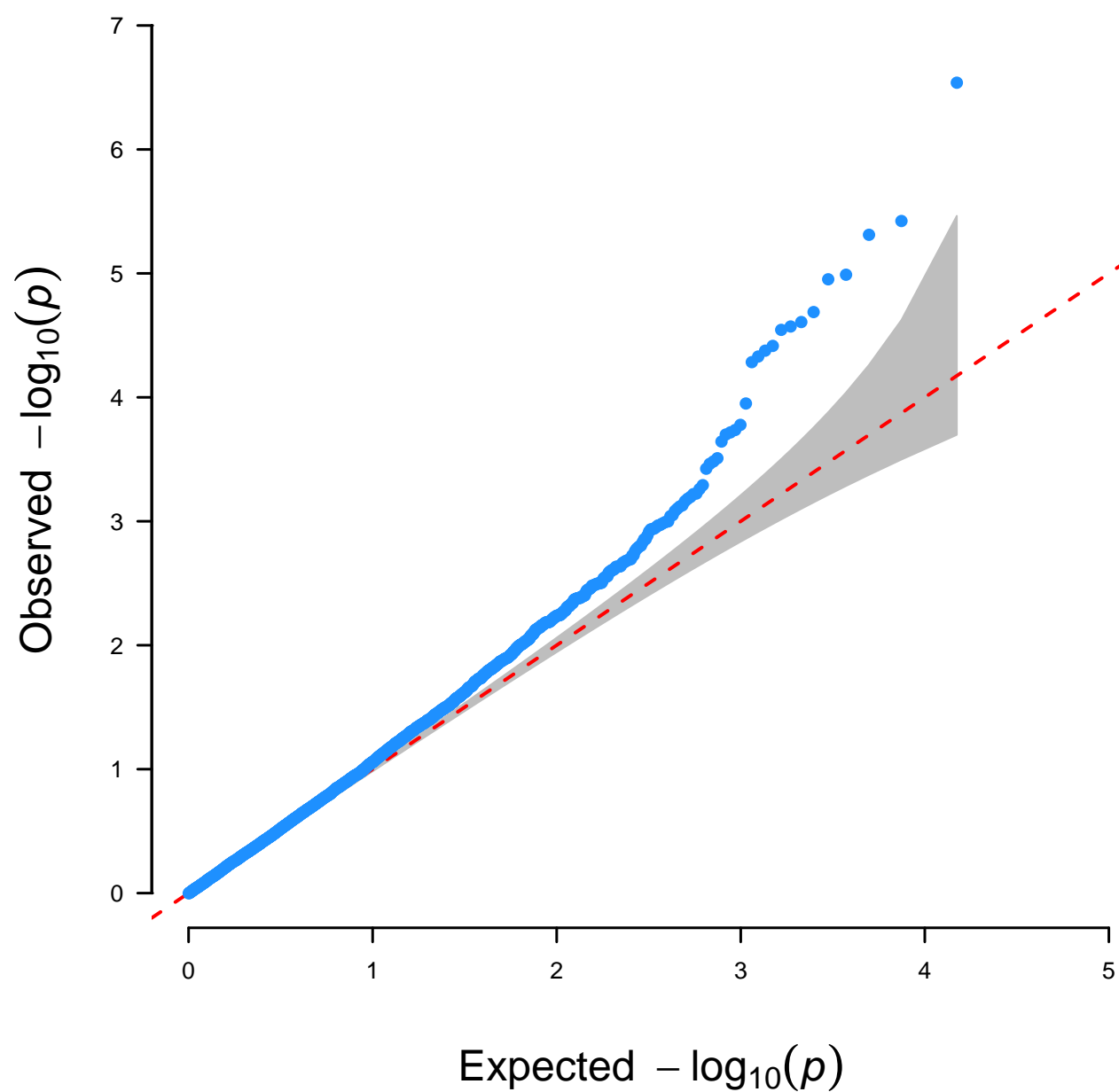**(b)**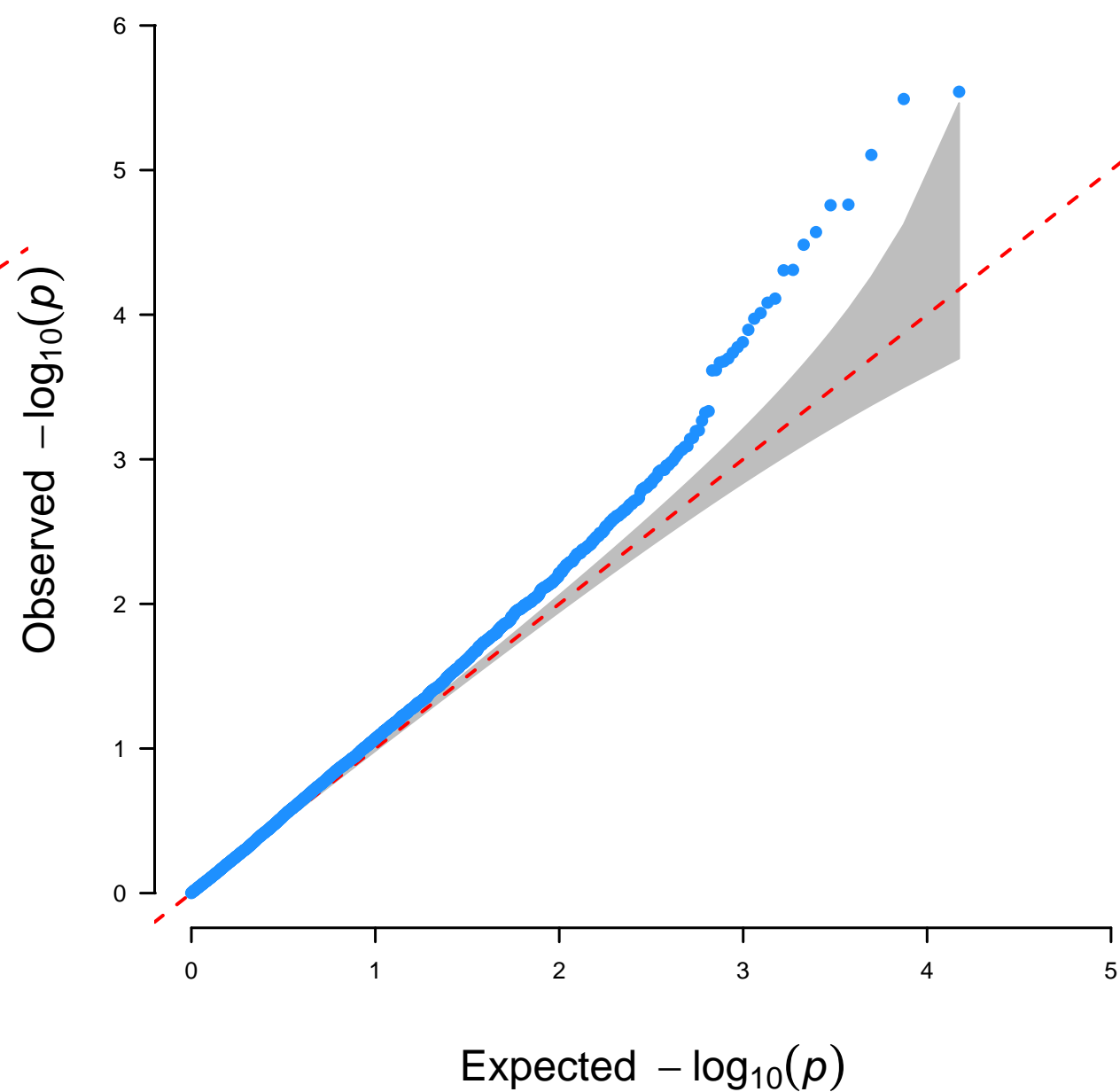**(c)**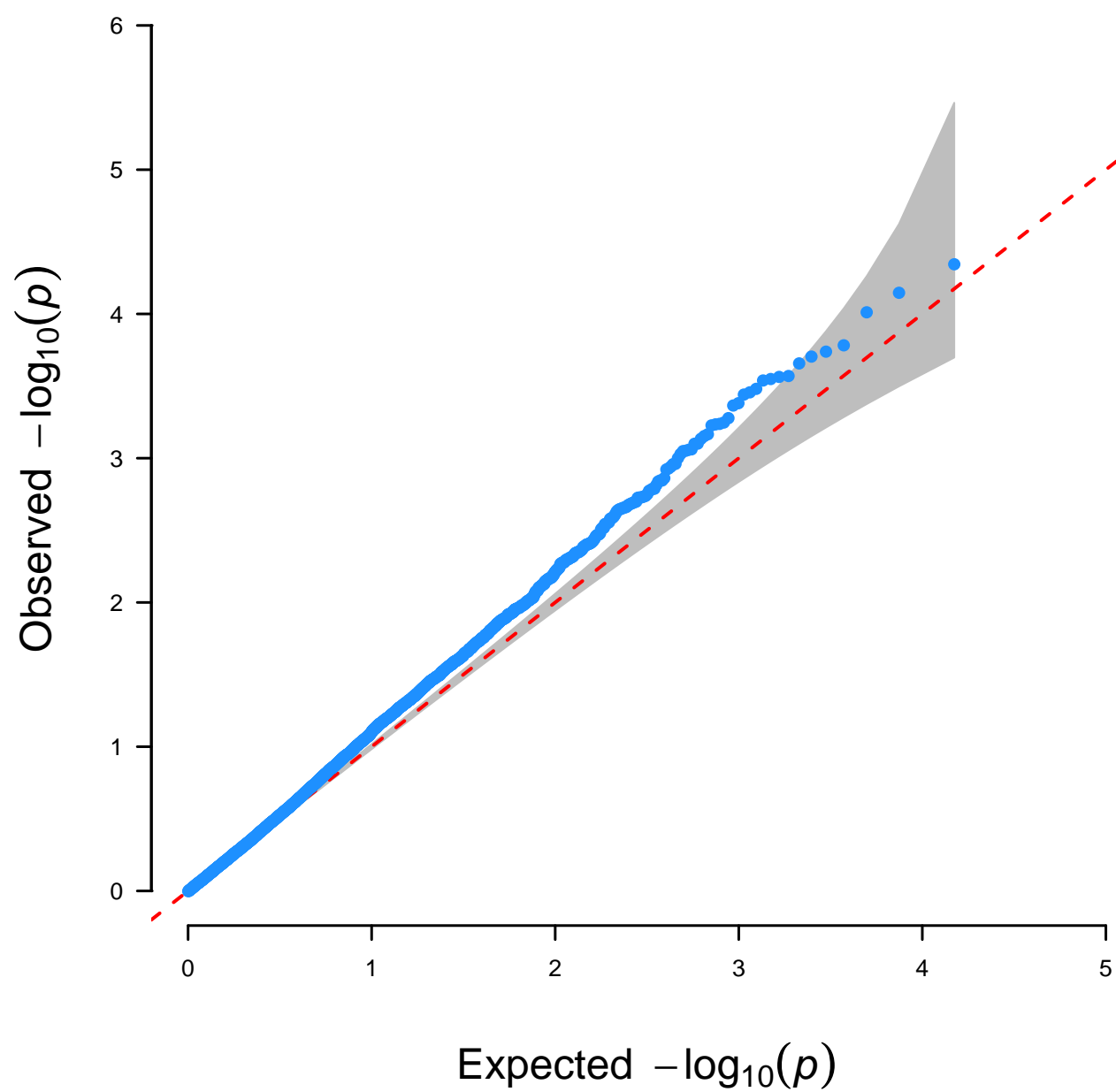**(d)**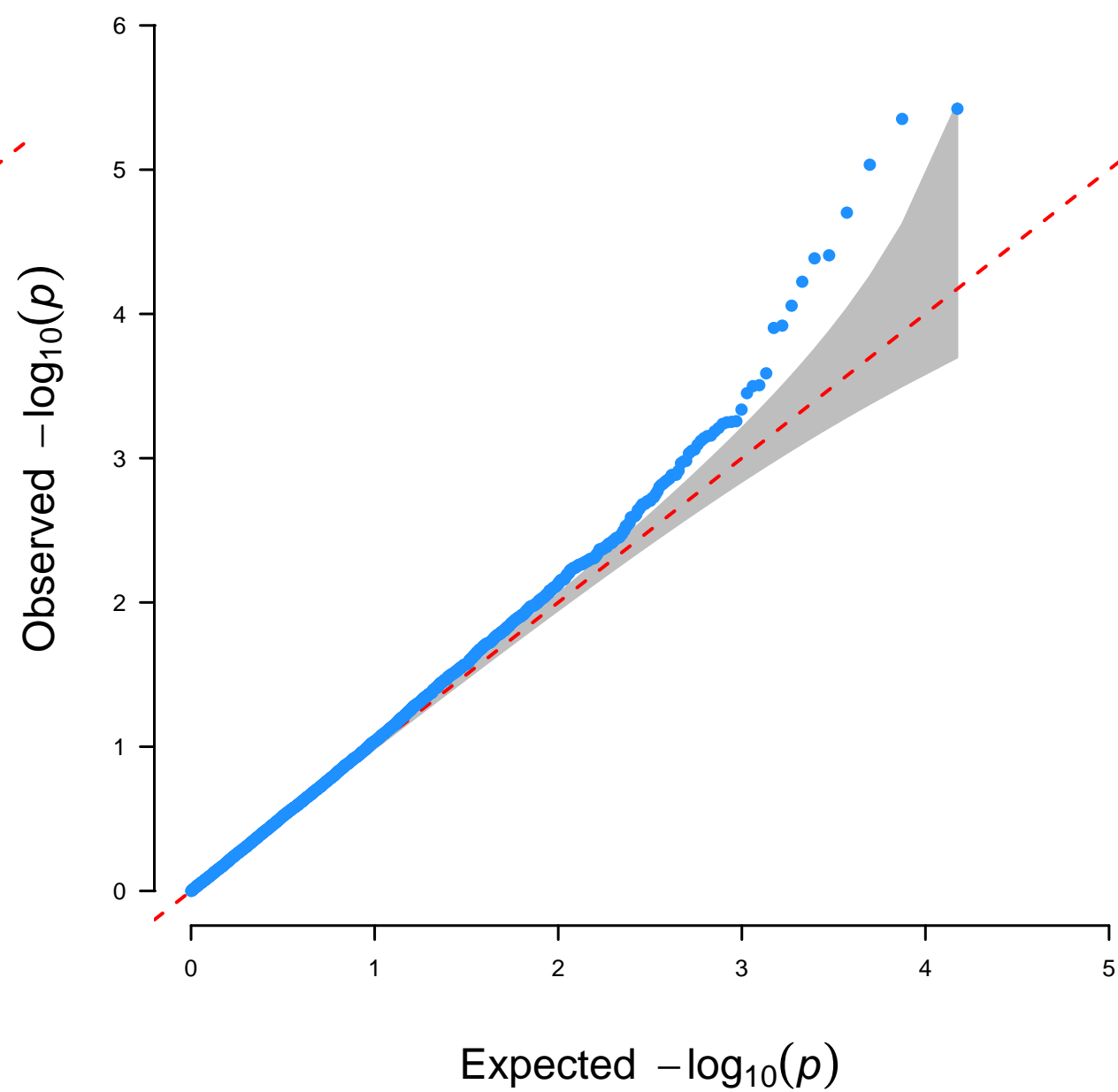

Supplement: Supplementary file 6 — Additional file 6: Figure S4. Quantile–quantile (QQ) plots of the association analyses for (a) IMF percentage, (b) TG content, (c) CHO content, and (d) PL content using linear mixed model. The grey areas in the QQ plots represent the 95% confidence intervals around the test statistics. [file 12711_2021_656_MOESM6_ESM.pdf]
